# An Immunosenescence-Related Gene Signature to Evaluate the Prognosis, Immunotherapeutic Response, and Cisplatin Sensitivity of Bladder Cancer

**DOI:** 10.1155/2022/2143892

**Published:** 2022-03-02

**Authors:** Ranran Zhou, Jingjing Liang, Hu Tian, Qi Chen, Cheng Yang, Cundong Liu

**Affiliations:** ^1^Department of Urology, The Third Affiliated Hospital of Southern Medical University, Guangzhou 510000, China; ^2^The Third School of Clinical Medicine, Southern Medical University, Guangzhou 510000, China; ^3^Department of Cardiology, Shunde Hospital of Southern Medical University, Foshan 528000, China

## Abstract

Immunosenescence refers to the immune system undergoing a series of degenerative changes with advancing age and is tightly associated with the initiation and progression of cancers. However, the immunosenescence-related genes as critical biomarkers for bladder cancer (BLCA) have not been systematically analyzed. We retrieved the immunosenescence-related genes from the public database and verified their association with hallmarks of immunosenescence based on The Cancer Genome Atlas (TCGA) cohort. Through gene pairing, Lasso, and univariate Cox regression, an 8-gene pair model was constructed to evaluate the overall survival of BLCA, which was then validated in the training cohort (*P* < 0.001, *n* = 396), two external validation cohorts (*P* < 0.05, *n* = 165; *P* < 0.001, *n* = 224), and local samples (*P* < 0.05, *n* = 10). We also downloaded the clinical information and gene expression matrices of other 32 different cancers from TCGA. The established model showed significant predictive value for the prognosis in 15 cancers (*P* < 0.05). The risk model could also serve as a promising predictor for immunotherapeutic response, which has been verified by the TIDE algorithm (*P* < 0.05), IMvigor210 dataset (*P* < 0.01, *n* = 298), and other two datasets correlated with immunotherapy (*P* < 0.05, *n* = 56; *P* = 0.17, *n* = 27). The TCGA dataset, in vitro cell experiments, and pan-cancer analysis displayed that the gene signature was associated with cisplatin sensitivity (*P* < 0.05). Overall, we proposed a novel immunosenescence-related gene signature to predict prognosis, immunotherapeutic response, and cisplatin sensitivity of BLCA, which were validated in different independent cohorts, local samples, and pan-cancer analyses.

## 1. Introduction

Bladder cancer (BLCA) is one of the predominant malignancies with high mortality and morbidity worldwide and is characterized by poor prognosis and high recurrence rates [1]. BLCA is mainly divided into nonmuscle invasive bladder cancer and muscle-invasive bladder cancer, which has less than 50% overall survival (OS) rates [2], based on the degree of cancer invasion. Tremendous progress in the treatment of BLCA has been made in recent years. Bacillus Calmette-Guerin (BCG) intravesical perfusion has been utilized for the treatment of BLCA since 1976 and serves as traditional management for the moment [3], demonstrating that BLCA is immunogenic. Recent studies display that BLCA is among the malignancies with the highest tumor mutational burden (TMB), and TMB was reported as the strongest predictor for immunotherapeutic effectiveness [4, 5]. On the whole, BLCA is prone to respond to chemotherapeutic reagents, including the newly proposed immune checkpoint inhibitors (ICIs) [6]. In addition to immunotherapy, neoadjuvant chemotherapy, especially cisplatin-based neoadjuvant chemotherapy, which has been recommended for the first-line treatment before radical cystectomy, dramatically improves the prognosis of BLCA [7]. Despite the advances in medical therapy, a large number of patients remain unresponsive to immunotherapy or chemotherapy and suffer unfavorable prognoses. Hence, seeking more accurate and reliable predictions for the prognosis and sensitivity to immunotherapy and chemosensitivity is a hot issue of much interest in BLCA.

With the rise and popularization of high-throughput sequencing, more and more biomarkers associated with the prognosis or drug response of BLCA have been discovered [8, 9]. The screened biomarkers not only helped to evaluate the clinical outcomes but also provided the clues to study the potential mechanisms of BLCA, such as ferroptosis [10], autophagy [11], and N6-methyladenosine (m6A) modification [12]. Nevertheless, the identification of the novel biomarkers with high accuracy and robustness is still meaningful, which could serve as promising clinical tools to guide the personalized treatment and the cut-in points to expound the correlated biological processes.

Most malignancies exhibit higher incidence rates in the elderly compared with the young, which is probably due in part to immunosenescence. The conception of immunosenescence was proposed by Walford in 1969 and referred to the immune system, including immune organs and immune cells, degenerated with aging [13]. The main hallmarks of immunosenescence include thymic involution, decreased T cell population and diversity, decreased antigen presentation ability of dendritic cells, attenuated phagocytosis of macrophages, and increased myeloid-derived suppressor cells (MDSCs), causing the decline of immune surveillance and cancer cell clearance [14]. The decreased immune surveillance promotes tumor immune escape, which directly leads to the proliferation, metastasis, and drug resistance of cancer cells. Although immunosenescence plays an essential role in cancer initiation and progression, the immunosenescence-related biomarkers have not been systematically analyzed in BLCA.

The present study collected the immunosenescence-related genes from the public database and verified their association with the hallmarks of immunosenescence in The Cancer Genome Atlas-Bladder Cancer (TCGA-BLCA) cohort, which was also set as the training dataset. We adopted a gene pair strategy to construct the risk signature to render the model applicable in different detection platforms [15]. Lasso, univariate Cox, and multivariate Cox regression analyses were used to identify the hub gene pairs associated with the OS in BLCA. The predictive ability of the risk model to immunotherapeutic and chemotherapeutic response was also detected. GSE13507, GSE32894, GSE5287, IMvogor210, GSE35640, GSE78220, pan-cancer analysis, in vitro cell experiments, and local samples were used for external validation.

## 2. Materials and Methods

### 2.1. Data Source and Processing

The senescence-related genes were retrieved from the Molecular Signatures Database (MSigDB, https://www.gsea-msigdb.org/gsea/msigdb/), and the immune-related genes were obtained from ImmPort (https://www.immport.org/), as shown in Supplementary Tables 1 and 2, respectively. The gene sets of the main hallmarks of immunosenescence, including senescence-associated secretory phenotype (SASP), mitochondrial biogenesis, glycolysis, and cellular response to reactive oxygen species (ROS), were also downloaded from MSigDB [16]. The transcriptional data with fragments per kilobase per million mapped reads (FPKM) format, corresponding clinical information, and genomic mutation data (varscan software) of the TCGA-BLCA cohort were downloaded from TCGA (https://tcga-data.nci.nih.gov/tcga/, accessed on August 20, 2021). The maftools package in R was used to calculate the TMB of each sample and to visualize the Top 30 genes with the highest mutational rate in the different subgroups. The RNA expression matrices and OS data of the other 32 cancers were also obtained from TCGA. GSE13507 [17], GSE32894 [18], GSE5287 [19], GSE35640 [20], and GSE78220 [21] were directly downloaded from the Gene Expression Omnibus (GEO, https://www.ncbi.nlm.nih.gov/geo/, accessed on August 21, 2021). The RNA expression data with FPKM format and clinical features of the IMvogor210 cohort were extracted from the IMvigor210CoreBiologies package in R software (version 3.6.3). We utilized R to conduct quality control, and the genes with average expression < 0.5 and the cases with <30 days' follow-up were excluded.

### 2.2. Clinical Sample Collection

The BLCA tissues from 10 patients undergoing partial/radical cystectomy were collected between November 2019 and July 2021 in the Third Affiliated Hospital of Southern Medical University. All the fresh BLCA samples were collected from the center of the tumor during surgery and stored in liquid nitrogen for RNA extraction. The patients undergoing preoperative chemotherapy, immunotherapy, and radiotherapy were not included in this study. The project has been approved by the Ethics Committee of the Third Affiliated Hospital of Southern Medical University (ID: 20211121), and the informed consent files were signed by all patients. The diagnosis of BLCA depends on the histopathological examination, and the detection of the tumor tissues' tumor-node-metastasis (TNM) stages was based on the eighth TNM staging system defined by the American Joint Commission on Cancer. Among the 10 patients, 1 patient was in TNM stage I, 4 patients were in TNM stage II, 3 patients were in TNM stage III, and 2 patients were in TNM stage IV.

### 2.3. Single-Sample Gene Set Enrichment Analysis (ssGSEA)

The ssGSEA was performed with the GSVA and the GSEABase packages with method specifications as “ssgsea,” “Gaussian,” and “abs.ranking=TRUE”, and the ssGSEA *Z*-score of each sample was calculated to quantify the enrichment level. The collected immunosenescence-related genes were set as the reference gene set.

### 2.4. Risk Model Construction

To make the risk model applicable in different detection platforms, we adopted the gene pair method to construct the model. We defined a gene combination form, “gene A | gene B,” as a gene pair. If the expression of gene A was higher than that of gene B, the pair would be considered 1; otherwise, it would be regarded as 0. When the amount of the gene pairs with values equal to 1 or 0 accounts for less than 20% of the total pairs, the pairs would be excluded. All the genes were cyclically singly paired, and a 0-or-1 matrix was established. Lasso and univariate Cox regression were performed to identify the significant gene pairs associated with OS of BLCA through the glmnet and the survival packages. In the univariate Cox analysis, *P* < 0.001 was thought to be significant. Subsequently, the gene pairs con-determined by Lasso and univariate Cox regressions were included in the multivariate Cox regression analysis with stepwise by the survminer package, and the risk model was ultimately constructed. The risk score evaluated with the risk model was defined as immunosenescence-related score (IRS). IRS was calculated using the following formula:

(1) $$\begin{array}{c} \text{IRS}=\overset{n}{\underset{i=1}{\sum }}{\text{Coeff}}_{i}*{\left(\text{gene pair}\right)}_{i}. \end{array}$$

### 2.5. Validation of the Risk Model

The Kaplan-Meier survival analysis with log-rank test was performed to detect the prognosis difference between high- and low-IRS patients through the survival package, and *P* < 0.05 was regarded to be significant. The optimal cut-off value was determined through X-tile software. The time-dependent receiver operating curves (ROCs) were drawn via the survivalROC package to evaluate the predictive ability of IRS to 1-, 3-, and 5-year OS rates and to compare the predictive ability of IRS and the risk clinicopathological traits, including age, gender, grade, stage, and TNM stage, after the transformation of all the continuous variables into binary variables.

### 2.6. Functional Enrichment Analysis

The Gene Ontology (GO) and Kyoto Encyclopedia of Genes and Genomes (KEGG) functional annotation were conducted via the clusterProfiler package in R with *P* < 0.05 and *Q* < 0.05 filtering. The enrichment analyses were visualized with the enrichplot and the ggplot2 packages. The Gene Set Enrichment Analysis (GSEA) was performed in GSEA desktop software (version 4.1.0, https://www.gsea-msigdb.org/gsea/) with 1,000 permutations and default parameters. The gene sets with a false discovery rate (FDR) < 0.25 and ∣normalized enrichment score (NES) | >1 were considered to be significant [22].

### 2.7. The Analyses of Immune Infiltration and Immunotherapeutic Response

The infiltration level of different immune cells among the samples from the TCGA-BLCA project was evaluated via CIBERSORT, which calculated the infiltration proportion of 22 kinds of immune cells [23]. The infiltration immune cell content difference between the high- and low-IRS groups was measured with the Wilcoxon signed-rank test. We also utilized the Tumor Immune Dysfunction and Exclusion (TIDE) algorithm to predict the response to ICIs of the patients from the TCGA-BLCA cohort [24] and conducted the Chi-squared test to detect the association with the IRS stratification. The IMvigor210 cohort, containing 298 patients with metastatic urothelial cancer (UC) undergoing atezolizumab (an anti-PD1 agent) treatment, were also used for external validation through Kaplan-Meier survival analysis, Wilcoxon signed-rank test, and Chi-squared test. GSE35640, including 56 patients with melanoma, and GSE78220, including 27 patients with melanoma, were selected to verify the predictive ability of IRS to immunotherapy in other malignancies via the Wilcoxon signed-rank test. The filtering threshold was set to *P* < 0.05.

### 2.8. The Evaluation of Chemotherapeutic Response

The sensitivity to the common chemotherapeutic reagents, such as cisplatin, doxorubicin, gemcitabine, methotrexate, and vinblastine, of the samples from the TCGA-BLCA project was quantified as half inhibitory concentration (IC50) through the pRRophetic package. Besides, the IRSs among the patients with different clinical statuses, including complete response (CR), partial response (PR), progression disease (PD), and stable disease (SD), were also compared. The Wilcoxon signed-rank test was adopted for difference detection, and *P* < 0.05 was significant.

### 2.9. Cell Culture and Treatment

Human BLCA cell line T24 was purchased from the Stem Cell Bank, Chinese Academy of Sciences (Shanghai, China), and maintained in McCoy's 5A Medium (Gibco, USA), which has been added with 100 U/ml penicillin, 100 *μ*g/ml streptomycin, and 10% fetal bovine serum (Gibco, USA), in a humidified atmosphere with 5% CO_2_ at 37°C. The cells were treated with 20 *μ*M cisplatin (Sigma-Aldrich, USA) for 24 hours before total RNA extraction [25].

### 2.10. Real-Time Quantitative PCR (RT-qPCR)

The total RNA of the human and cell samples was isolated with the TRIzol/chloroform method (TRIzol, Invitrogen, USA) after the homogenization of human samples, following the manufacturer's instruction. The cDNA was synthesized with the PrimeScript RT reagent Kit (Takara, Japan), and the RT-qPCR was performed with the SYBR Premix Ex Taq II reagent (Takara, Japan) based on the Applied Biosystems 7300 Real-Time PCR System (Applied Biosystems, USA). GAPDH was chosen as the interreference to normalize the data, and the 2-*ΔΔ*Ct method was used to calculate the mRNA expression value. The statistical analysis was performed with Student's *t*-test. The primers utilized in this study are shown in Supplementary Table 3.

### 2.11. Statistical Analysis

The statistical analyses of the present study were conducted in R software (version 3.6.3). The data were presented as the mean ± standard deviation (mean ± SD) or *n* (%). The Wilcoxon signed-rank test was used to compare the difference in different groups if not otherwise specifically stated. The Pearson Chi-squared test was performed to analyze the association of categorical data, which was visualized via the ggplot2 package. The ROCs as the performance evaluation method for binary classification ability were drawn via the pROC package. ^∗^*P* < 0.5,  ^∗∗^*P* < 0.01, and^∗∗∗^*P* < 0.001.

## 3. Results

### 3.1. Identification of the Immunosenescence-Related Genes

**Figure 1:**
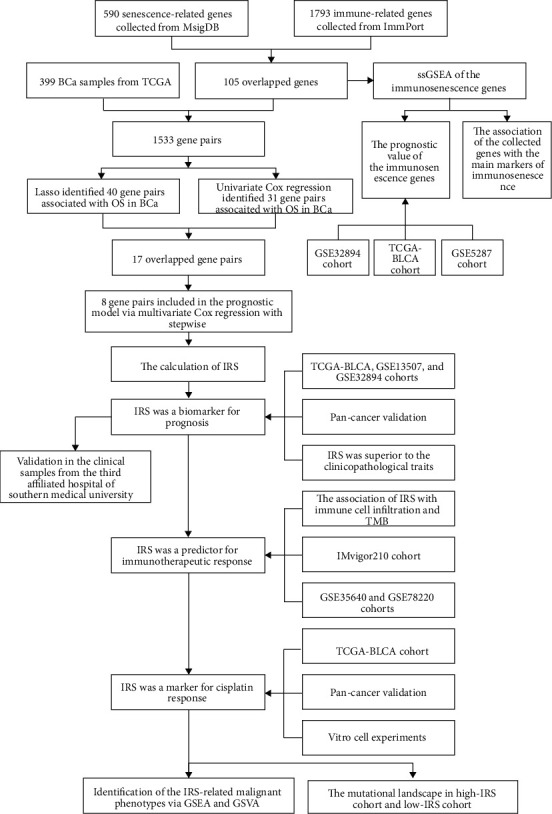
The work flowchart of the whole study.

**Figure 2:**
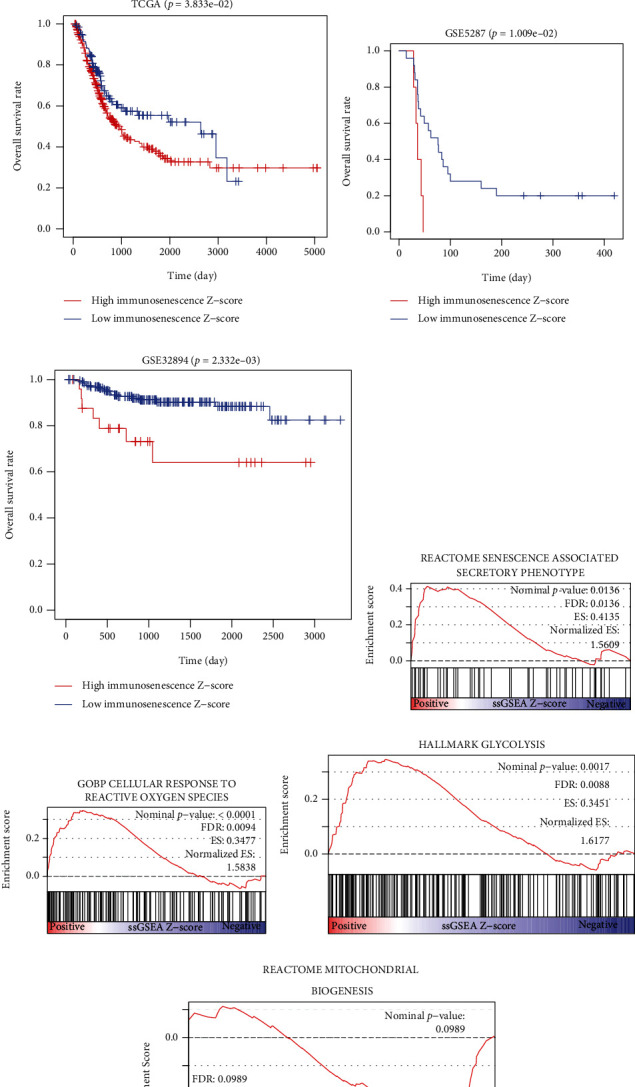
The identification of immunosenescence-related genes. (a) 105 overlapped genes from the senescence-related genes and the immune-related genes. (b) The distribution of the ssGSEA *Z*-scores of the senescence-related gene sets, immune-related gene set, and the potential immunosenescence-related gene set in the TCGA-BLCA cohort. (c–e) The high ssGSEA *Z*-score heralded a worse prognosis in the TCGA-BLCA cohort (c), GSE5287 cohort (d), and GSE32894 cohort (e). (f–i) The association between the ssGSEA *Z*-score and the main hallmarks of immunosenescence, including senescence-associated secretory phenotype (f), cellular response to reactive oxygen species (g), glycolysis (h), and mitochondrial biogenesis (i). (j) The association between the ssGSEA *Z*-score and the immunosenescence's marker genes. TCGA: The Cancer Genome Atlas; BLCA: bladder cancer; ssGSEA: single-sample gene set enrichment analysis. ^∗^*P* < 0.05,  ^∗∗^*P* < 0.01, and^∗∗∗^*P* < 0.001.

The workflow of the present study is shown in Figure 1. First, 105 common genes con-determined by the 590 senescence-related genes from MSigDB and 1793 immune-related genes from ImmPort were identified as the potential immunosenescence-related genes (Figure 2(a)). The ssGSEA was implemented to calculate the enrichment level of the retrieved immunosenescence-related gene set among the 396 BLCA samples from the TCGA-BLCA project (Figure 2(b)). It was found that the BLCA samples with high ssGSEA *Z*-score exhibited worse OS in the TCGA-BLCA cohort (*P* < 0.05, Figure 2(c)), implying that the collected gene set was significantly associated with the prognosis. The prognosis value of the gene set was also validated in the patients with BLCA from the GSE5287 cohort (*P* < 0.05, *n* = 30, Figure 2(d)) and GSE32894 cohort (*P* < 0.01, *n* = 224, Figure 2(e)). GSEA indicated that SASP (Figure 2(f)), cellular response to ROS (Figure 2(g)), and glycolysis (Figure 2(h)) were positively associated with the ssGSEA *Z*-score, while mitochondrial biogenesis (Figure 2(i)) was negatively associated with the ssGSEA *Z*-score. The increase of SASP, cellular response to ROS, and glycolysis and the decrease of mitochondrial biogenesis have been reported as the main hallmarks of immunosenescence [16]. Besides, the association between the ssGSEA *Z*-score and the known immunosenescence marker genes was also detected (Figure 2(j)). Overall, the immunosenescence-related genes collected from the public databases could reflect the immunosenescence process to some extent and were chosen for further analysis.

### 3.2. Risk Model Construction

**Figure 3:**
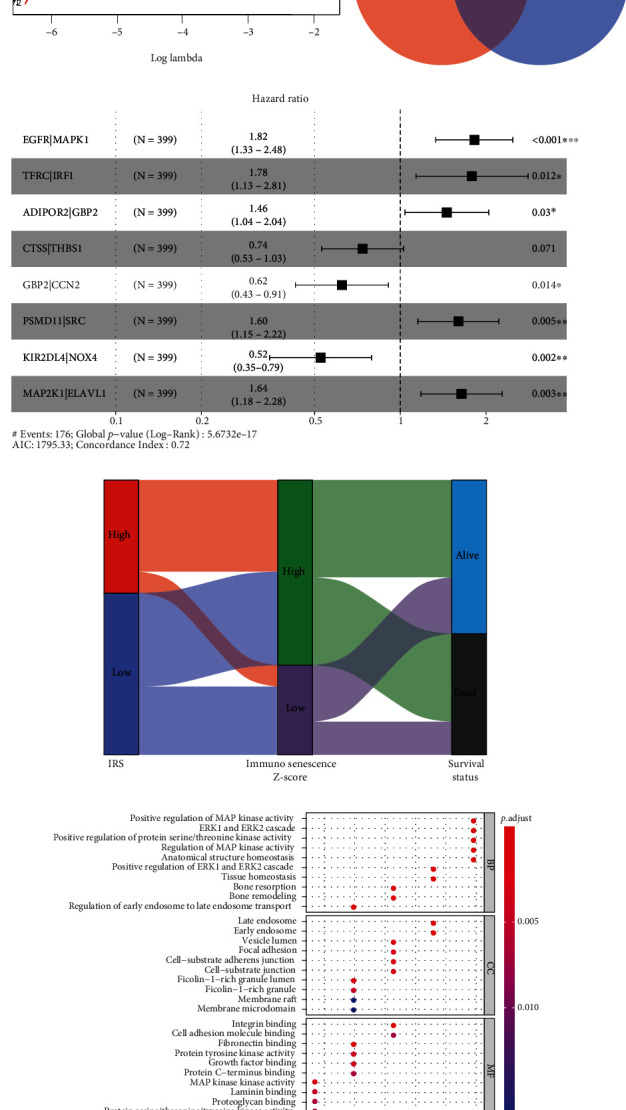
The construction of the risk model. (a, b) Lasso regression identified 40 gene pairs associated with the OS. (c) 17 gene pairs were con-determined via the Lasso regression and univariate Cox analysis. (d) The forest plot indicated that 8 gene pairs were ultimately included in the risk model through multivariate Cox regression with stepwise. (e) The distribution of the IRS stratification, ssGSEA *Z*-score stratification, and survival statuses in the TCGA-BLCA cohort. (f, g) GO (f) and KEGG (g) functional annotation of the 15 genes comprising the risk signature. OS: overall survival; IRS: immunosenescence-related score; TCGA: The Cancer Genome Atlas; BLCA: bladder cancer; ssGSEA: single-sample gene set enrichment analysis; GO: Gene Ontology; KEGG: Kyoto Encyclopedia of Genes and Genomes.

After the cyclical pairing, a total of 1533 gene pairs were established. Lasso regression identified that 40 of 1533 gene pairs were significantly associated with the OS (Figures 3(a) and 3(b)). At the same time, 31 gene pairs were determined by univariate Cox regression with *P* < 0.001 filterings. 17 gene pairs were con-determined by these dimension-reduction methods (Figure 3(c)), 8 of which were ultimately included in the risk model via multivariate Cox analysis with stepwise (Figure 3(d)). The IRS was calculated as follows: IRS = 0.599∗(EGFR | MAPK1) + 0.579∗(TFRC | IRF1) + 0.376∗(ADIPOR2 | GBP2)–0.305∗(CTSS | THBS1)–0.472∗(GBP2 | CCN2) + 0.469∗(PSMD11 | SRC)–0.645∗(KIR2DL4 | NOX4) + 0.495∗(MAP2K1 | ELAVL1), where (gene A  |  gene B) represented a gene pair. According to the optimal cut-off value detected by X-tile, which was equal to 2.90, all the patients in the training cohort and the external validation cohorts were divided into the high- and low-IRS subgroups. The Sankey plot displayed the association among IRSs, ssGSEA *Z*-scores, and the survival statuses in the TCGA-BLCA project (Figure 3(e)). Figures 3(f) and 3(g) indicated the GO and KEGG functional annotation of the 15 genes comprising the risk signature, respectively, where some important pathways both associated with immunosenescence and tumorigenesis, such as chemical carcinogenesis-reactive oxygen species and PD-L1 expression and PD-1 checkpoint pathway in cancer, were significantly enriched.

### 3.3. IRS Is a Robust Biomarker for Prognosis Prediction

**Figure 4:**
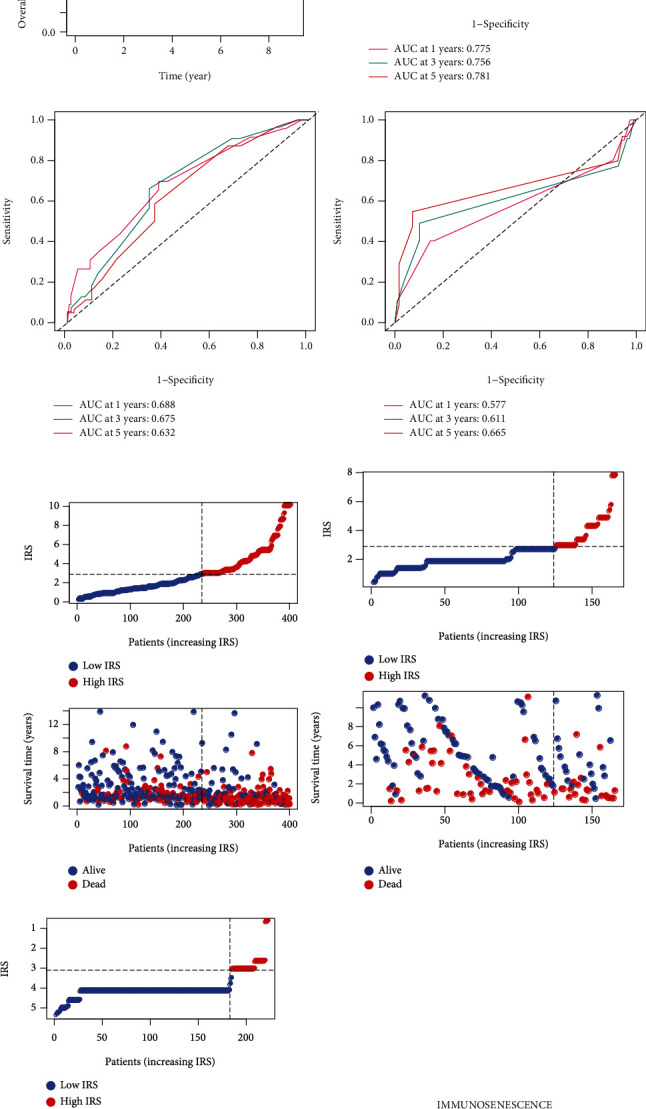
Validation of risk model's prognostic value. (a–c) Kaplan-Meier survival plots displayed that the patients with high IRS exhibited worse prognosis in the TCGA-BLCA cohort (a), GSE13507 cohort (b), and GSE32894 cohort (c). The cut-off was set to 2.90. (d–f) The ROCs for the evaluation of IRS to 1-, 3-, and 5-year OS in the TCGA-BLCA cohort (d), GSE13507 cohort (e), and GSE32894 cohort (f). (g–i) The distribution of IRS and survival statuses among the BLCA patients from the TCGA-BLCA cohort (g), GSE13507 cohort (h), and GSE32894 cohort (i). (j–l) The subjects with high IRS were more likely to exhibit immunosenescence statuses no matter in the TCGA-BLCA cohort (j), GSE13507 cohort (k), or GSE32894 cohort (l). OS: overall survival; IRS: immunosenescence-related score; TCGA: The Cancer Genome Atlas; BLCA: bladder cancer; ROC: receiver operating curve.

We validated the robustness of the risk signature in the training dataset, 2 external validation datasets from the public database, local BLCA samples, and pan-cancer analysis. The baseline information of the training dataset (TCGA-BLCA) and the 2 external validation dataset (GSE13507 and GSE32894) is displayed in Table 1. Kaplan-Meier survival analyses showed that the BLCA patients with high IRS suffered a lower OS rate in the TCGA-BLCA cohort (*P* < 0.001, *n* = 396, Figure 4(a)), GSE13507 cohort (*P* < 0.05, *n* = 165, Figure 4(b)), and GSE32894 cohort (*P* < 0.001, *n* = 224, Figure 4(c)). The time-dependent ROCs for the evaluation of 1-, 3-, and 5-year OS in TCGA-BLCA (Figure 4(d)), GSE13507 (Figure 4(e)), and GSE32894 (Figure 4(f)) verified the predictive value. Besides, with the increase of IRS, more deaths were observed, as shown in the scatter plots (Figure 4(g)–4(i)). The patients with high IRS in the TCGA-BLCA (Figure 4(j)), GSE13507 (Figure 4(k)), and GSE32894 (Figure 4(l)) were more likely to exhibit immunosenescence statuses via the GSEA. The expression level of the 15 genes in the risk model in BLCA samples and adjacent normal samples is shown in Supplementary Figure 1, and their predictive performance for the OS of BLCA in the TCGA-BLCA cohort (Supplementary Figure 2), GSE13507 cohort (Supplementary Figure 3), and GSE32894 cohort (Supplementary Figure 4) was measured via Kaplan-Meier survival analyses.

Next, the tumor samples of the 10 patients with BLCA from the local hospital were collected for experimental validation. The 10 patients were divided into the TNM stage I-II and TNM stage III-IV groups, and the gene expression value was measured via RT-qPCR. The expression difference of the 15 genes in the risk signature between TNM stage I-II and TNM stage III-IV is shown in Supplementary Figure 5a, and the statistical method was Welch's *t*-test. According to the detected gene expression value, the IRS of each patient was calculated (Supplementary Figure 5b). The patients in TNM stage III-IV have higher IRSs compared with those in TNM stage I-II through Welch's *t*-test (*P* < 0.05, Supplementary Figure 5c), which partly proved the reliability of the risk model.

**Figure 5:**
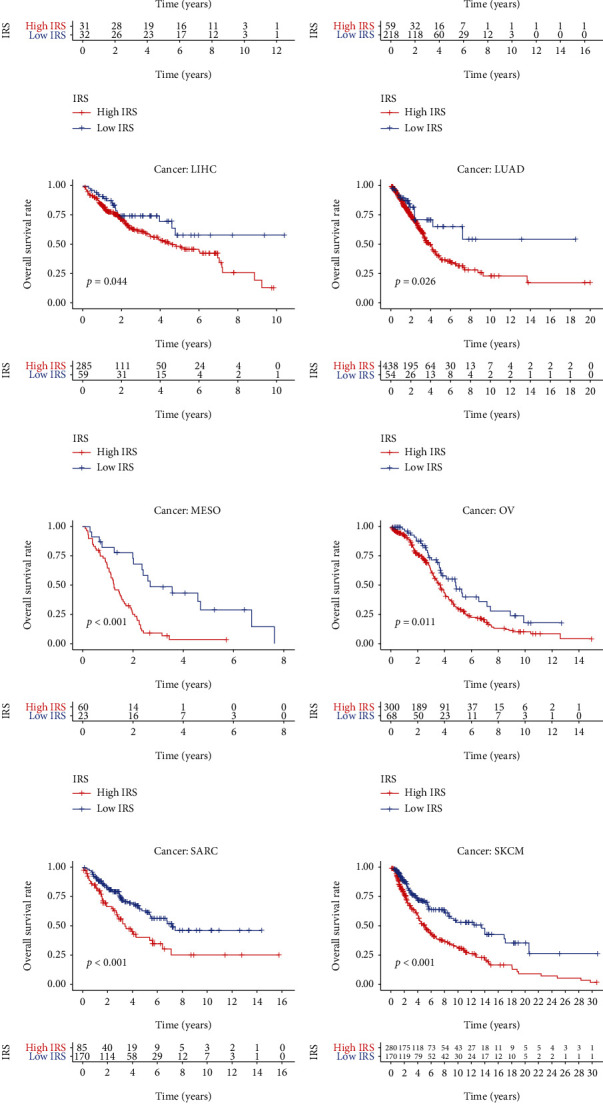
Pan-cancer analysis indicated that IRS is capable of predicting the OS of adrenocortical carcinoma (a), breast invasive carcinoma (b), cervical squamous cell carcinoma and endocervical adenocarcinoma (c), head and neck squamous cell carcinoma (d), chromophobe renal cell carcinoma (e), papillary renal cell carcinoma (f), hepatocellular carcinoma (g), lung adenocarcinoma (h), mesothelioma (i), ovarian serous cystadenocarcinoma (j), sarcoma (k), skin cutaneous melanoma (l), stomach adenocarcinoma (m), thyroid carcinoma (n), and endometrial carcinoma (o). IRS: immunosenescence-related score; OS: overall survival.

To confirm whether the established signature could serve as a predictor for the prognosis in pan-cancer, we downloaded the datasets of other 32 cancers from TCGA, and the detailed information is shown in Table 2. As shown in Figure 5, the IRS was a significant biomarker for the prognosis in 15 different cancers, implying that IRS was a powerful predictor in pan-cancer.

### 3.4. The Clinical Association of IRS

**Figure 6:**
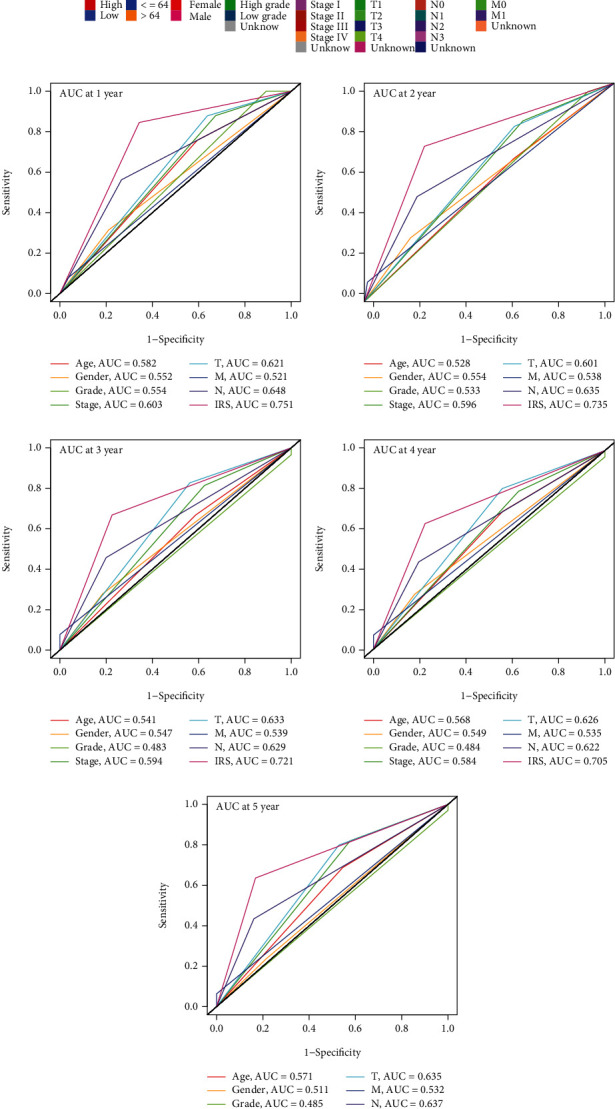
The clinical association of IRS. (a) The Chi-squared test between IRS and the risk clinicopathological features. (b–f) IRS was superior to the risk clinicopathological parameters in the prediction of 1-year (b), 2-year (c), 3-year (d), 4-year (e), and 5-year (f) OS. IRS: immunosenescence-related score; OS: overall survival.

The Chi-squared test indicated that IRS was significantly associated with age (*P* < 0.05), gender (*P* < 0.01), tumor grade (*P* < 0.001), TNM stage (*P* < 0.001), and pathological T stage (*P* < 0.001) based on the TCGA-BLCA project, as shown in Figure 6(a). After transforming all the factors into binary variables, we compared the predictive ability of the clinicopathological parameters and IRS to OS using ROC analyses. The optimal cut-off of age was detected by the X-tile software, and the patients over 64 years old suffered the most significantly worse OS rates among the subjects in the TCGA-BLCA cohort (*P* < 0.001). The areas under curves (AUCs) of IRS were all higher than those of the risk clinical features in the 1-year (AUC = 0.751, Figure 6(b)), 2-year (AUC = 0.735, Figure 6(c)), 3-year (AUC = 0.721, Figure 6(d)), 4-year (AUC = 0.705, Figure 6(e)), and 5-year (AUC = 0.734, Figure 6(f)) OS evaluation. IRS was also an independent risk factor for OS among the BLCA patients from the TCGA-BLCA cohort through the univariate (hazard ratio, HR; confidence interval, CI; HR = 4.30, 95%CI = 2.57-7.17, *P* < 0.01) and the multivariate (HR = 3.79, 95% CI = 2.18-6.61, *P* < 0.01) analyses (Table 3).

### 3.5. IRS Is a Promising Tool to Evaluate the Immunotherapeutic Response

**Figure 7:**
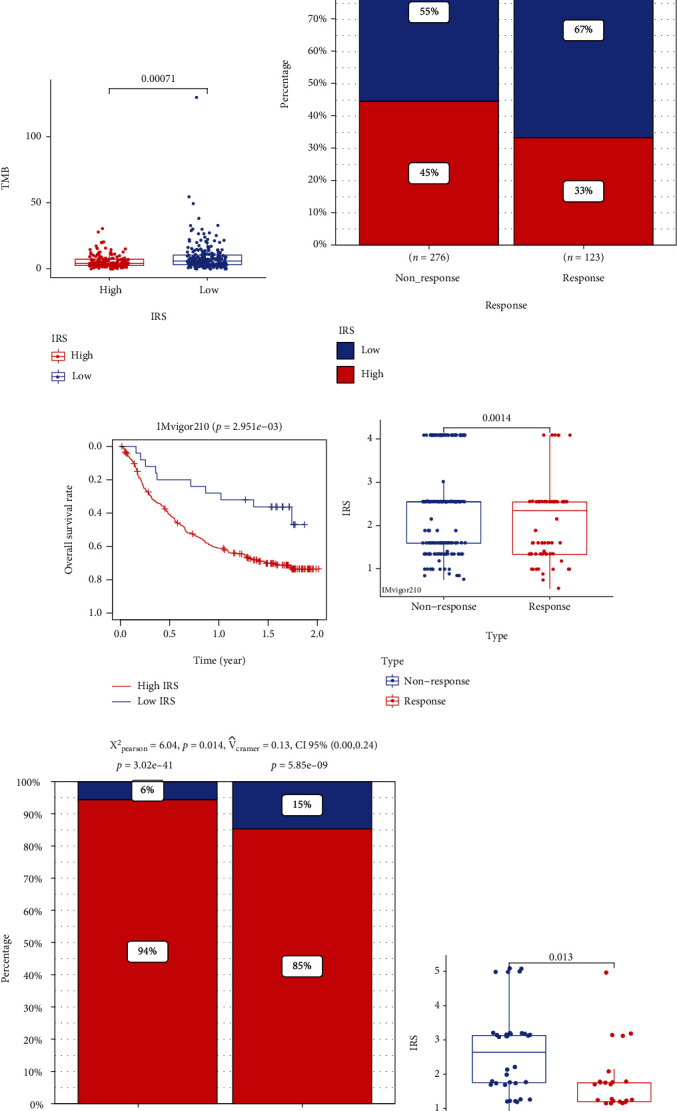
IRS is also a predictor for immunotherapeutic response. (a) The infiltration level of the 22 immune cells in the high- and low-IRS subgroups. (b) IRS was negatively associated with TMB. (c) TIDE algorithm indicated that the subjects with low IRS were more likely to respond to immunotherapeutic reagents in the TCGA-BLCA project. (d) IRS was a significant biomarker to evaluate the OS among patients with metastatic urothelial cancer undergoing anti-PD1 treatment. (e, f) Wilcoxon signed-rank test (e) and Pearson Chi-squared test (f) displayed that the subjects with low IRS were more likely to respond to immunotherapeutic reagents in the IMvogor210 cohort. (g, h) The difference of IRSs between the subjects with melanoma showing response to immunotherapy and the subjects showing no response to immunotherapy from the GSE35640 cohort (g) and GSE78220 cohort (h). IRS: immunosenescence-related score; TMB: tumor mutational burden; TCGA: The Cancer Genome Atlas; BLCA: bladder cancer. ^∗^*P* < 0.05,  ^∗∗^*P* < 0.01, and^∗∗∗^*P* < 0.001.

Compared with the low-IRS group, the high-IRS group exhibited a higher infiltration level of resting memory CD4 T cells (*P* < 0.05), M0 macrophages (*P* < 0.001), M2 macrophages (*P* < 0.05), activated mast cells (*P* < 0.01), and neutrophils (*P* < 0.05) and the lower infiltration proportion of naïve B cells (*P* < 0.01), memory B cells (*P* < 0.01), CD8 T cells (*P* < 0.001), activated memory CD4 T cells (*P* < 0.001), follicular helper T cells (*P* < 0.01), and activated NK cells (*P* < 0.05), indicating the tremendous changes of tumor immune microenvironment between high- and low-IRS patients (Figure 7(a)). Besides, the low-IRS patients harbored higher TMB (*P* < 0.001, Figure 7(b)), suggesting that the low-IRS subjects were more likely to benefit from immunotherapy, and the TIDE algorithm verified the assumption (*P* < 0.05, Figure 7(c)). Next, we validated the predictive ability of IRS in different independent cohorts receiving immunotherapy. It was found that the patients with metastatic UC showing high IRS exhibited poorer survival rates in the IMvigor210 cohort (*P* < 0.01, Figure 7(d). Compared with the subjects with no response to atezolizumab, the patients with high sensitivity to atezolizumab had lower IRSs via the Wilcoxon signed-rank test (*P* < 0.01, Figure 7(e)) and Chi-squared test (*P* < 0.05, Figure 7(f)). In addition to the BLCA patients, 2 melanoma cohorts who had received immunotherapy, GSE35640 and GSE78220, were also used for validation. Figures 7(g) and 7(h) displayed that the patients with resistance to immunotherapy had higher IRSs, redemonstrating the potential of IRS to evaluate the immunotherapy sensitivity.

The expression difference of the 15 genes in the risk signature between the responsive subjects and nonresponsive subjects from the IMvigor210 cohort is shown in Supplementary Figure 6a. Supplementary Figure 6b displays the predictive value of each variable to the OS of the IMvigor210 cohort.

### 3.6. IRS Can Predict Cisplatin Sensitivity

**Figure 8:**
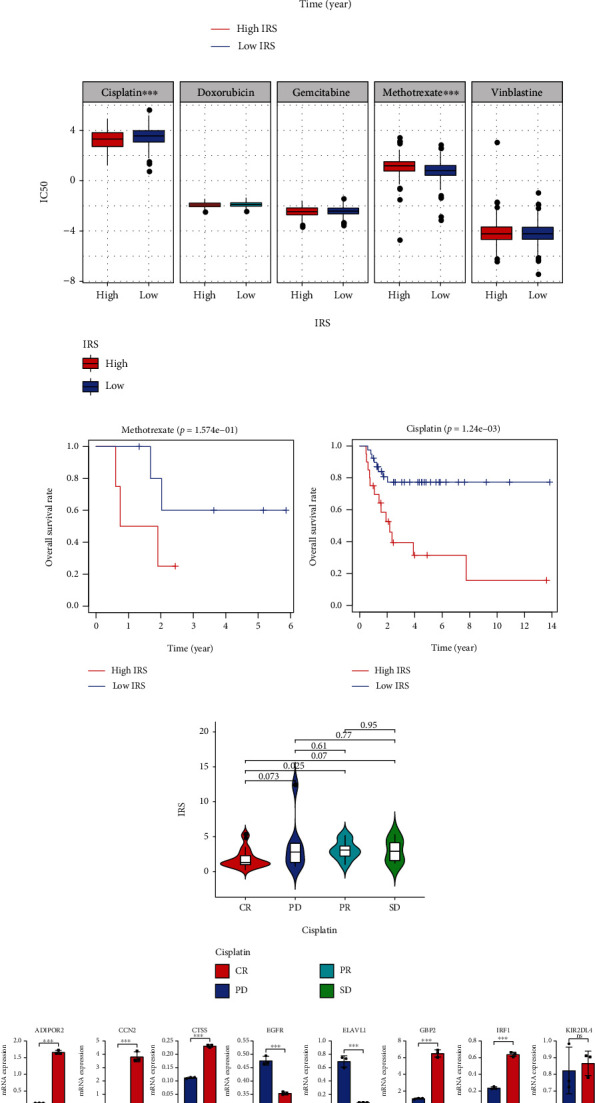
IRS can evaluate the cisplatin sensitivity. (a) IRS could distinguish the high-risk subjects among the patients with BLCA receiving chemotherapy. (b) IRS was significantly associated with the IC50 of cisplatin and methotrexate. (c) IRS was not a significant prognosis predictor for the patients receiving methotrexate treatment. (d) IRS could distinguish the high-risk subjects among the patients receiving cisplatin treatment. (e) The association between IRS and the clinical response to cisplatin in the TCGA-BLCA cohort. (f) The expression level of the genes comprising the risk signature in the T24 cells treated with/without cisplatin. IRS: immunosenescence-related score; IC50: half inhibitory concentration; TCGA: The Cancer Genome Atlas; BLCA: bladder cancer. ^∗^*P* < 0.05,  ^∗∗^*P* < 0.01, and^∗∗∗^*P* < 0.001.

A total of 109 patients receiving chemotherapy were extracted from the TCGA-BLCA project, and IRS could still discriminate against the high-risk patients (*P* < 0.001, Figure 8(a)).  Figure 8(b) indicates that IRS was significantly associated with the IC50 of cisplatin (*P* < 0.001) and methotrexate (*P* < 0.001). However, the Kaplan-Meier survival plots showed that IRS was not a significant prognosis predictor among the patients receiving methotrexate treatment (*P* > 0.05, Figure 8(c)), but IRS could significantly distinguish the high-risk subjects receiving cisplatin treatment (*P* < 0.01, Figure 8(d)). The IRS level of the patients with CR, PD, PR, and SD to cisplatin treatment is shown in Figure 8(e), and a significant difference between the CR and PR groups was observed (*P* < 0.05). However, it should be stated that most cases in the TCGA-BLCA cohort received chemotherapeutic agent combination therapy. Therefore, a series of in vitro cell experiments to reconfirm the association was conducted, and the T24 cells with cisplatin treatment were used for external validation. As shown in Figure 8(f), the level of ADIPOR2 (*P* < 0.001), CCN2 (*P* < 0.001), CTSS (*P* < 0.001), GBP2 (*P* < 0.001), IRF1 (*P* < 0.001), MAP2K1 (*P* < 0.01), TFRC (*P* < 0.001), and THBS1 (*P* < 0.01) was obviously increased after cisplatin treatment, while the level of EGFR (*P* < 0.001), ELAVL1 (*P* < 0.001), MAPK1 (*P* < 0.001), NOX4 (*P* < 0.001), and SRC (*P* < 0.01) was markedly decreased, suggesting that most of the genes in the risk signature have a tight relationship with the pharmacology of cisplatin.

**Figure 9:**
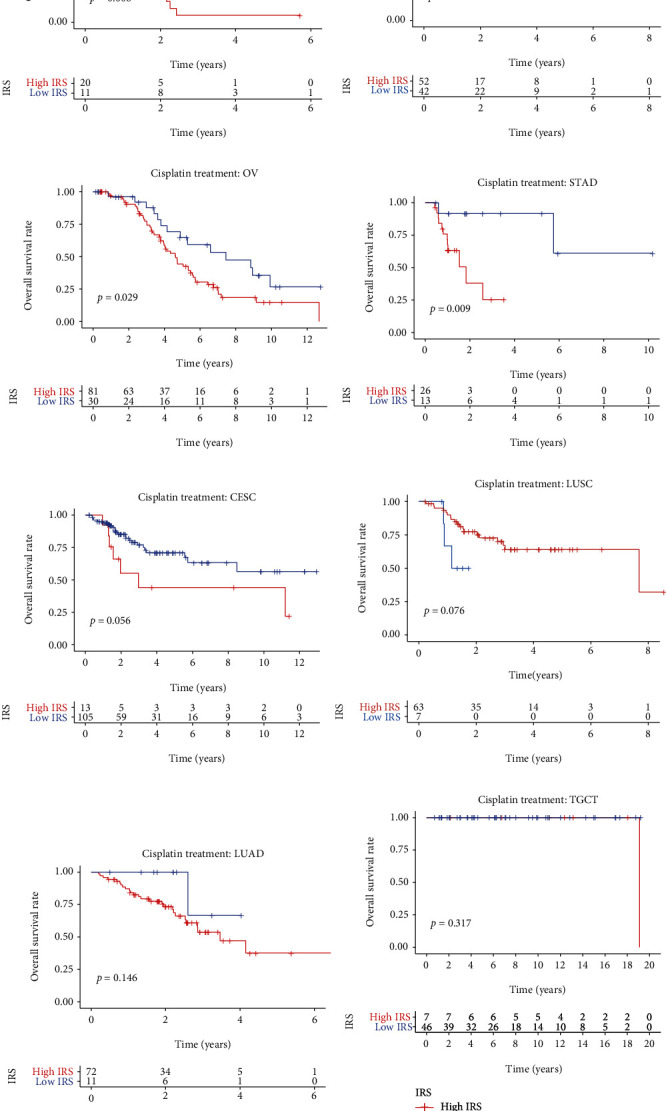
The predictive performance of IRS to cisplatin sensitivity in mesothelioma (a), head and neck squamous cell carcinoma (b), ovarian serous cystadenocarcinoma (c), stomach adenocarcinoma (d), cervical squamous cell carcinoma and endocervical adenocarcinoma (e), lung squamous cell carcinoma (f), lung adenocarcinoma (g), and testicular germ cell tumors (h). IRS: immunosenescence-related score; MESO: mesothelioma; HNSC: head and neck squamous cell carcinoma; OV: ovarian serous cystadenocarcinoma; STAD: stomach adenocarcinoma; CESC: cervical squamous cell carcinoma and endocervical adenocarcinoma; LUSC: lung squamous cell carcinoma; LUAD: lung adenocarcinoma; TGCT: testicular germ cell tumors.

Pan-cancer analysis was also used for validation, and the other 8 types of cancer, which contained >20 patients receiving cisplatin treatment in the TCGA project, were selected (Table 2). IRS showed powerful predictive capability for the prognosis among the cases receiving cisplatin treatment with mesothelioma (MESO, *P* < 0.01, Figure 9(a)), head and neck squamous cell carcinoma (HNSC, *P* < 0.05, Figure 9(b)), ovarian serous cystadenocarcinoma (OV, *P* < 0.05, Figure 9(c)), and stomach adenocarcinoma (STAD, *P* < 0.01, Figure 9(d)). Figures 9(e)–9(h) displayed the predictive ability of IRS in cervical squamous cell carcinoma and endocervical adenocarcinoma (CESC, *P* > 0.05), lung squamous cell carcinoma (LUSC, *P* > 0.05), lung adenocarcinoma (LUAD, *P* > 0.05), and testicular germ cell tumors (TGCT, *P* > 0.05), respectively, and the heterogeneity and limited sample size might account for the nonsignificance. Overall, IRS was a reliable predictor for cisplatin sensitivity, which has been validated in different cohorts and cell experiments.

### 3.7. The Mutational Landscape in High- and Low-IRS Patients

**Figure 10:**
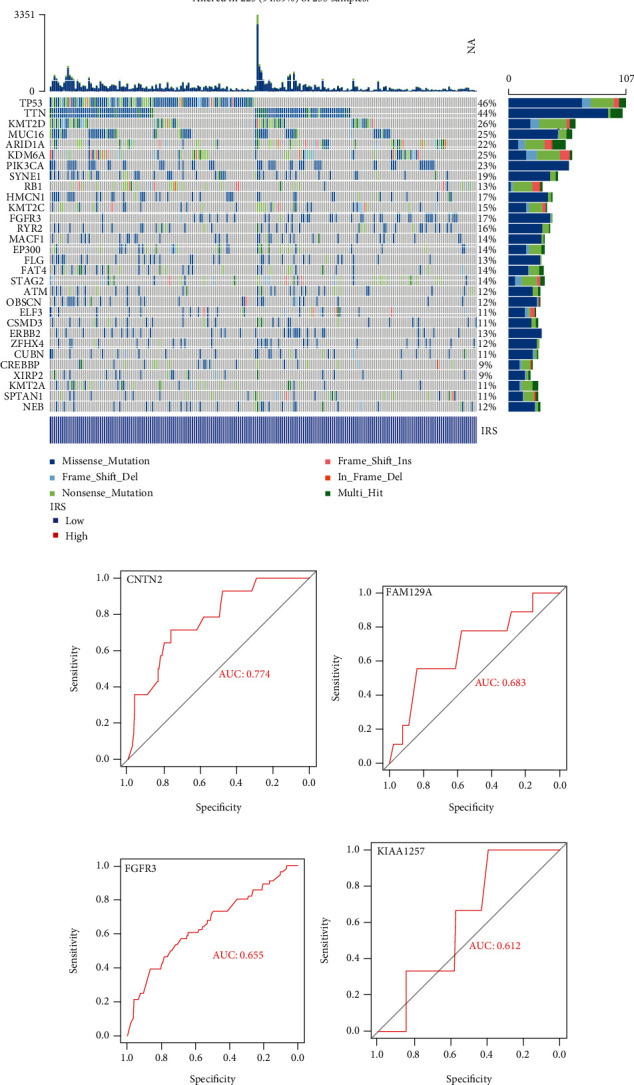
The mutational landscape between the high- and low-IRS groups. (a, b) The Top 30 genes with the highest mutational rates in the high-IRS (a) and low-IRS (b) patients. (c–i) IRS could predict the mutational statuses of CNTN2 (c), FAM129A (d), FGFR3 (e), KIAA1257 (f), NCF1 (g), SIGMAR1 (h), and STAG2 (i). IRS: immunosenescence-related score.

Since the IRS was tightly associated with TMB, we then explored the mutational divergence between high- and low-IRS patients based on the TCGA-BLCA project. The Top 30 genes with the highest mutational rate in the high- and low-IRS subgroups are shown in Figures 10(a) and 10(b), respectively. We found that IRS could predict the mutational statuses of CNTN2 (AUC = 0.774, Figure 10(c)), FAM129A (AUC = 0.683, Figure 10(d)), FGFR3 (AUC = 0.655, Figure 10(e)), KIAA1257 (AUC = 0.612, Figure 10(f)), NCF1 (AUC = 0.619, Figure 10(g)), SIGMAR1 (AUC = 0.840, Figure 10(h)), and STAG2 (AUC = 0.622, Figure 10(i)) with high efficacy, implying that the mutation of these genes was correlated with IRS.

## 4. Discussion

**Figure 11:**
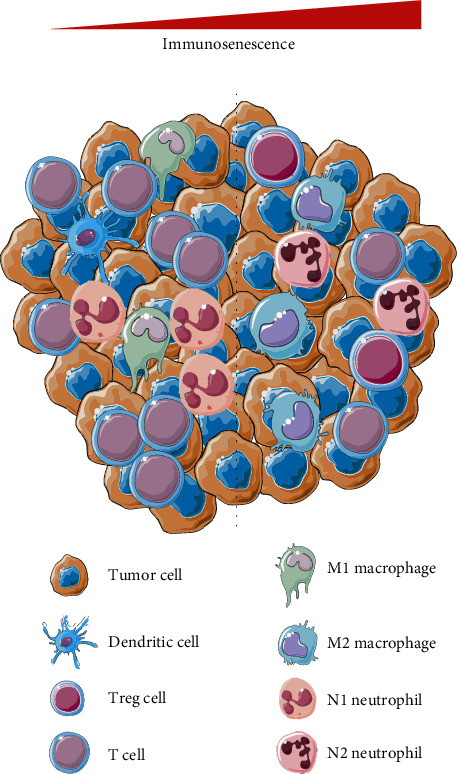
Immunosenescence is tightly associated with the immunosuppressive atmosphere in the tumor microenvironment.

With the population aging, the prevalence and mortality of BLCA increase gradually in many regions. Immunosenescence is accepted as one of the leading causes of tumorigenesis, which directly promotes the evasion of immune surveillance and the immunosuppressive atmosphere in tumor microenvironment [26, 27]. With the advance of immunosenescence, the infiltration level of dendritic cells, T cells, M1 macrophages, and N1 neutrophils decreases, while the infiltration abundance of the immunosuppressive cells, such as Treg cells, M2 macrophages, and N2 neutrophils, increases (Figure 11). The depletion of functional T cells, the attenuation of antigen-presenting ability, and the accumulation of immunosuppressive cells lead to unfavorable drug treatment effectiveness and poor prognosis [28]. Hence, the identification of the immunosenescence-related biomarkers is important and meaningful, but no immunosenescence-related gene model has been reported in BLCA to date.

In this study, we retrieved the immunosenescence-related genes from the MSigDB and ImmPort and verified the association of the collected genes with the known hallmarks of immunosenescence via ssGSEA and GSEA. A sum of 105 immunosenescence-related genes was screened. Subsequently, we adopted a gene pair method to construct the risk signature, and the 105 genes were cyclically singly paired. Lasso, univariate Cox, and multivariate Cox regressions identified an 8-gene pair model to evaluate the OS based on the TCGA-BLCA project. Here, we defined the risk score calculated by the model as IRS. GSE13507, which included 165 BLCA samples, GSE32894, which included 224 BLCA samples, 10 BLCA samples collected from the local hospital, and pan-cancer analysis were used for external validation to confirm the prognostic value of IRS. The Wilcoxon signed-rank test displayed that IRS was significantly associated with TMB and immune infiltration level in the tumor microenvironment, which was calculated via CIBERSORT. Therefore, we then explored whether IRS could serve as a predictor for the immunotherapeutic response. The validation in the TCGA-BLCA, IMvigor210 cohort, GSE35640 cohort, and GSE78220 cohort proved the assumption. IRS was also a marker for cisplatin treatment, which has been validated in the cohort receiving cisplatin treatment from the TCGA-BLCA project, T24 cells treated with cisplatin, and pan-cancer analyses. The mutational landscape in the high- and low-IRS groups was also detected.

The more accurate and reliable prediction for the prognosis and treatment response is always one of the hottest issues in BLCA. Many predictive gene models have been proposed, providing useful clinical tools for clinicians and BLCA patients [29, 30]. However, the number of the risk model used for the evaluation of prognosis, immunotherapeutic response, and chemosensitivity at the same time is limited to date. A predictive model applied to multiple different scenarios at the same time is more practical and more portable. Besides, we adopted the gene pair strategy to construct the signature, which means that the risk model does not depend on the definite gene expression value and could be applied for different detection platforms, including RT-qPCR, RNA-seq, microarray, and NanoString. Overall, the proposed model in this study was a promising and practical tool for BLCA and can be used to guide personalized treatment.

Some genes were first reported as biomarkers for BLCA. For instance, GBP2 was significantly upregulated in the BLCA samples with low malignancy (Supplementary Figure 5), and the high expression GBP2 heralded a favorable prognosis (Supplementary Figures 4 and 6). Godoy et al. reported that GBP2 was significantly associated with the favorable prognosis in 766 patients with breast cancer, implying that GBP2 was a tumor suppressor [31]. In general, the established signature helped to identify novel biomarkers in BLCA.

However, some shortcomings should not be neglected. First, this study was designed as a retrospective and multicenter research due to the limited financial support, and a prospective, randomized, and double-blind clinical trait would be more beneficial to verify the clinical usefulness. Second, experimental validation, such as transfection assays and tumor-forming experiments in athymic mice, should be conducted to clarify the biological functions of the screened novel biomarkers.

## 5. Conclusions

To sum up, a novel immunosenescence-related gene signature was developed to estimate the prognosis, immunotherapeutic response, and cisplatin sensitivity in BLCA, which has been validated in different independent cohorts, local BLCA samples, in vitro cell experiments, and pan-cancer analyses.

## Figures and Tables

**Table 1:** The baseline information of the BLCA patients.

Parameters	TCGA (*n* = 396)	GSE13507 (*n* = 165)	GSE32894 (*n* = 224)
Survival status			
Alive	243 (61.3%)	96 (58.1%)	199 (88.8%)
Dead	153 (38.6%)	69 (41.8%)	25 (11.1%)
Follow-up (day)	778.19 ± 814.38	1451.45 ± 1127.70	1196.98 ± 767.38
Age	67.84 ± 10.53	65.18 ± 11.93	69.43 ± 11.28
Gender			
Female	104 (26.2%)	30 (18.1%)	61 (27.2%)
Male	292 (73.7%)	135 (81.8%)	163 (72.7%)
Grade			
Low	18 (4.5%)	105 (63.6%)	—
High	375 (94.7%)	60 (36.3%)	—
Unknown	3 (0.8%)	0 (0.0%)	—
BLCA TNM stages			
I	2 (0.5%)	—	—
II	124 (31.3%)	—	—
III	138 (34.8%)	—	—
IV	130 (32.8%)	—	—
Unknown	2 (0.5%)	—	—
pT stage			
T0	1 (0.2%)	0 (0.0%)	0 (0.0%)
Ta	0 (0.0%)	23 (13.9%)	110 (49.1%)
T1	3 (0.7%)	81 (49.0%)	63 (28.1%)
T2	113 (28.5%)	31 (18.7%)	43 (19.1%)
T3	190 (47.9%)	19 (11.5%)	7 (3.1%)
T4	57 (14.3%)	11 (6.6%)	1 (0.4%)
Unknown	32 (8.0%)	0 (0.0%)	0 (0.0%)
M stage			
M0	189 (47.7%)	158 (95.7%)	—
M1	10 (2.5%)	7 (4.2%)	—
Unknown	197 (49.7%)	0 (0.0%)	—
pN stage			
N0	229 (57.8%)	149 (90.3%)	27 (12.0%)
N1	44 (11.1%)	8 (4.8%)	3 (1.3%)
N2	75 (18.9%)	6 (3.6%)	10 (4.4%)
N3	7 (1.7%)	1 (0.6%)	0 (0.0%)
Unknown	41 (10.3%)	1 (0.6%)	184 (82.1%)
Risk stratification			
High	164 (41.1%)	41 (24.8%)	38 (17.1%)
Low	235 (58.8%)	124 (75.1%)	183 (82.8%)
IRS	2.90 ± 2.49	2.39 ± 1.27	1.99 ± 0.67

TCGA: The Cancer Genome Atlas; IRS: immunosenescence-related score; BLCA: bladder cancer; TNM: tumor-node-metastasis.

**Table 2:** The detailed information of the samples across 33 cancer types.

Cancer type	Full name	Tumor samples	Patients receiving cisplatin treatment	IRS (mean ± SD)
ACC	Adrenocortical carcinoma	79	2	1.33 ± 1.04
BLCA	Bladder urothelial carcinoma	399	113	2.90 ± 2.49
BRCA	Breast invasive carcinoma	1098	2	1.85 ± 0.92
CESC	Cervical squamous cell carcinoma and endocervical adenocarcinoma	306	118	1.40 ± 1.13
CHOL	Cholangiocarcinoma	36	5	1.40 ± 1.18
COAD	Colon adenocarcinoma	458	0	1.18 ± 0.61
DLBC	Lymphoid neoplasm diffuse large B-cell lymphoma	48	2	0.77 ± 0.49
ESCA	Esophageal carcinoma	162	14	2.47 ± 1.63
GBM	Glioblastoma multiforme	167	6	3.11 ± 1.21
HNSC	Head and neck squamous cell carcinoma	502	94	3.18 ± 1.95
KICH	Chromophobe renal cell carcinoma	65	0	2.20 ± 1.09
KIRC	Clear cell renal cell carcinoma	531	0	2.34 ± 1.50
KIRP	Papillary renal cell carcinoma	289	1	1.48 ± 1.06
LAML	Acute myeloid leukemia	151	0	0.58 ± 0.26
LGG	Brain lower grade glioma	525	0	2.81 ± 1.19
LIHC	Hepatocellular carcinoma	373	4	2.12 ± 1.31
LUAD	Lung adenocarcinoma	515	83	1.93 ± 1.24
LUSC	Lung squamous cell carcinoma	501	70	3.06 ± 1.68
MESO	Mesothelioma	86	31	2.09 ± 1.37
OV	Ovarian serous cystadenocarcinoma	379	111	1.44 ± 0.78
PAAD	Pancreatic adenocarcinoma	178	2	1.50 ± 0.77
PCPG	Pheochromocytoma and paraganglioma	183	0	2.13 ± 0.92
PRAD	Prostate adenocarcinoma	496	0	1.60 ± 0.94
READ	Rectum adenocarcinoma	167	0	1.14 ± 0.60
SARC	Sarcoma	263	2	1.45 ± 1.06
SKCM	Skin cutaneous melanoma	471	9	1.32 ± 0.89
STAD	Stomach adenocarcinoma	375	39	1.23 ± 0.81
TGCT	Testicular germ cell tumors	156	53	1.16 ± 0.79
THCA	Thyroid carcinoma	510	0	1.31 ± 0.82
THYM	Thymoma	119	6	0.92 ± 0.53
UCEC	Endometrial carcinoma	544	20	1.12 ± 0.66
UCS	Uterine carcinosarcoma	56	8	1.93 ± 0.93
UVM	Uveal melanoma	80	0	0.88 ± 0.37

IRS: immunosenescence-related score; SD: standard deviation.

**Table 3:** Univariate and multivariate Cox analyses of the risk model.

Parameters	Univariate Cox		Multivariate Cox	
	HR (95% CI)	*P* value	HR (95% CI)	*P* value
Age (≤64 vs. >64)	1.42 (0.82-2.44)	0.20	1.11 (0.64-1.95)	0.70
Gender (female vs. male)	1.58 (0.94-2.66)	0.08	1.16 (0.68-1.99)	0.59
Grade (low vs. high)	3.65 (0.50-26.53)	0.20	0.99 (0.12-7.85)	0.99
Stage (I-II vs. III-IV)	2.26 (1.15-4.44)	0.02	1.18 (0.29-4.78)	0.82
T (T 1-2 vs. T 3-4)	2.41 (1.26-4.61)	0.01	1.57 (0.43-5.71)	0.49
N (N0 vs. N1-3)	2.34 (1.45-3.78)	<0.01	1.72 (0.98-3.02)	0.06
M (M0 vs. M1)	2.55 (1.02-6.40)	0.05	1.16 (0.43-3.12)	0.77
IRS (low vs. high)	4.30 (2.57-7.17)	<0.01	3.79 (2.18-6.61)	<0.01

HR: hazard ratio; CI: confidence interval; IRS: immunosenescence-related score.

## Data Availability

The data used to support the findings of this study are available from The Cancer Genome Atlas (TCGA, https://portal.gdc.cancer.gov/), GEO (https://www.ncbi.nlm.nih.gov/geo/), MSigDB (https://www.gsea-msigdb.org/gsea/msigdb/), and ImmPort (https://www.immport.org/), and the code would be supplied from the corresponding author upon request.

## Supplementary Materials

Supplementary 1 Supplementary Table 1: the collected senescence-related genes.

Supplementary 2 Supplementary Table 2: the collected immune-related genes.

Supplementary 3 Supplementary Table 3: the primer sequence utilized in this study.

Supplementary 4 Supplementary Figure 1: the expression difference of the 15 genes in the risk signature between adjacent normal and BLCA tissues in the TCGA-BLCA cohort (a), GSE13507 cohort (b), and GSE32894 cohort (c).

Supplementary 5 Supplementary Figure 2: the predictive value of the 15 genes to OS in the TCGA-BLCA cohort.

Supplementary 6 Supplementary Figure 3: the predictive value of the 15 genes to OS in the GSE13507 cohort.

Supplementary 7 Supplementary Figure 4: the predictive value of the 15 genes to OS in the GSE32894 cohort.

Supplementary 8 Supplementary Figure 5: validation of the prognostic value of IRS in the local cohort. (a) The expression level of the 15 genes in the risk signature in the subjects with BLCA in stage I-II and the subjects with BLCA in stage III-IV. (b) The calculated IRS of each patient. (c) The patients in stage III-IV exhibited higher IRS levels compared with those in stage I-II.

Supplementary 9 Supplementary Figure 6: the expression divergence of the 15 genes in the signature between the patients with high immunotherapeutic sensitivity and the patients with low immunotherapeutic sensitivity (a) and the prognostic value of each gene in the IMvigor210 cohort (b).
