# Supplementary material for: An Immunosenescence-Related Gene Signature to Evaluate the Prognosis, Immunotherapeutic Response, and Cisplatin Sensitivity of Bladder Cancer
Source: Dis Markers. 2022 Mar 2;2022:2143892. doi: 10.1155/2022/2143892 (PMC8915927; doi:10.1155/2022/2143892)
Supplement: Supplementary 1 — Supplementary Table 1: the collected senescence-related genes. [file 2143892.f1.pdf]

Supplementary Table 1 The collected senescence-related genes
E2F1
E2F3
NF1
PTEN
AKT1
BCL2
EGFR
HSP90AA1
IGF1R
KRAS
MYC
PPP2CA
PRKCA
RB1
TEP1
TERF1
TERT
TNKS
TP53
XRCC5
XRCC6
ACSS2
ATP5MC3
CA4
CALB1
COL1A1
COL3A1
COL4A5
CX3CL1
DIABLO
FABP3
GHITM
NDUFB11
NREP
TFRC
UQCRFS1
UQCRQ
ADIPOR2
ANXA3
ANXA5
APOD
B2M
C1QA
C1QB
C1QC
C3
C4A
CLIC4
CLU
CTSS

DCLK1
DERL1
EFCAB14
EFEMP1
FCGR2A
FCGR2B
GBP2
GFAP
GNS
GPNMB
GSTA1
H1-2
HBA1
HCST
HLA-G
IL33
JCHAIN
LAPTM5
LGALS3
LITAF
LYZ
MGST1
MPEG1
MSN
MT1F
NDRG1
NPC2
PCSK6
PSMD11
PTGES3
RASA3
RNF213
S100A4
S100A6
SERPING1
SGK1
SPP1
TMED10
TXNIP
VAT1
VWF
ALDH1A1
BMI1
CCN4
CCNB1
CDC25B
CKS1BP7
E2F4
EGR1
ID1
LAMA1

LDB2
MARCKS
ALDH1A3
AOPEP
CCN2
CCND1
CD44
CDKN1A
CDKN1C
CDKN2A
CDKN2B
CDKN2D
CITED2
CLTB
COL1A2
CREG1
CRYAB
CXCL14
CYP1B1
EIF2S2
ESM1
F3
FILIP1L
FN1
GSN
GUK1
HBS1L
HPS5
HSPA2
HTATIP2
IFI16
IFNG
IGFBP1
IGFBP2
IGFBP3
IGFBP4
IGFBP5
IGFBP6
IGFBP7
IGSF3
ING1
IRF5
IRF7
ISG15
MAP1LC3B
MAP2K3
MDM2
MMP1
NDN
NME2
NRG1

OPTN
PEA15
RAB13
RAB31
RAB5B
RABGGTA
RAC1
RBL2
RGL2
RHOB
RRAS
S100A11
SERPINB2
SERPINE1
SMPD1
SMURF2
SOD1
SPARC
STAT1
TES
TFAP2A
TGFB1I1
THBS1
TNFAIP2
TNFAIP3
TSPYL5
VIM
ABL1
AKT3
ARG2
ARNTL
ATM
ATR
BCL2L12
BCL6
BGLAP
BMPR1A
BRCA2
CALR
CDK6
CGAS
CHEK1
CHEK2
CTC1
DNAJA3
ECRG4
EEF1E1
ERCC1
FBXO5
FOXM1
FZR1

H2AX
HMGA1
HMGA2
HRAS
ICAM1
ID2
ING2
KAT6A
KIR2DL4
LIMS1
LMNA
MAGEA2
MAGEA2B
MAP2K1
MAP3K3
MAPK14
MAPKAPK5
MARCHF5
MIF
MIR10A
MIR146A
MIR17
MIR188
MIR20B
MIR21
MIR217
MIR22
MIR34A
MIR543
MIR590
MME
MNT
MORC3
MTOR
NEK4
NEK6
NOX4
NPM1
NSMCE2
NUAK1
NUP62
OPA1
PAWR
PDCD4
PLA2R1
PLK2
PML
PNPT1
PRELP
PRKCD
PRKDC

PRMT6
RBL1
ROMO1
RSL1D1
SIRT1
SLC30A10
SMC5
SMC6
SPI1
SRF
TBX2
TBX3
TERC
TERF2
TP63
TWIST1
ULK3
VASH1
WNT1
WNT16
WRN
YBX1
YPEL3
ZKSCAN3
ZMIZ1
ZMPSTE24
ZNF277
ZNF354A
CISD2
COMP
COQ7
DDC
EDN1
GBA
GHRHR
HYAL2
IDE
INPP5D
LEP
LRRK2
MSH2
MSH6
NR5A1
PRDM2
RAD54B
RAD54L
RNF165
SEC63
TFCP2L1
TH
TREX1

ACD
AGO1
AGO3
AGO4
ANAPC1
ANAPC10
ANAPC11
ANAPC15
ANAPC16
ANAPC2
ANAPC4
ANAPC5
ANAPC7
ASF1A
CABIN1
CBX2
CBX4
CBX6
CBX8
CCNA1
CCNA2
CCNE1
CCNE2
CDC16
CDC23
CDC26
CDC27
CDK2
CDK4
CDKN1B
CDKN2C
CEBPB
CXCL8
E2F2
EED
EHMT1
EHMT2
EP400
ERF
ETS1
ETS2
EZH2
FOS
H1-0
H1-1
H1-3
H1-4
H1-5
H2AB1
H2AC14
H2AC18

H2AC19
H2AC20
H2AC4
H2AC6
H2AC7
H2AC8
H2AJ
H2AZ1
H2AZ2
H2BC1
H2BC10
H2BC11
H2BC12
H2BC13
H2BC14
H2BC15
H2BC17
H2BC21
H2BC3
H2BC4
H2BC5
H2BC6
H2BC7
H2BC8
H2BC9
H2BS1
H2BU1
H3-3A
H3-3B
H3-4
H3C1
H3C10
H3C11
H3C12
H3C13
H3C14
H3C15
H3C2
H3C3
H3C4
H3C6
H3C7
H3C8
H4-16
H4C1
H4C11
H4C12
H4C13
H4C14
H4C15
H4C2

H4C3
H4C4
H4C5
H4C6
H4C8
H4C9
HIRA
IFNB1
IL1A
IL6
JUN
KAT5
KDM6B
LMNB1
MAP2K4
MAP2K6
MAP2K7
MAP3K5
MAP4K4
MAPK1
MAPK10
MAPK11
MAPK3
MAPK7
MAPK8
MAPK9
MAPKAPK2
MAPKAPK3
MDM4
MINK1
MIR24-1
MIR24-2
MOV10
MRE11
NBN
NFKB1
PHC1
PHC2
PHC3
POT1
RAD50
RBBP4
RBBP7
RELA
RING1
RNF2
RPS27A
RPS6KA1
RPS6KA2
RPS6KA3
SCMH1

SP1
STAT3
SUZ12
TERF2IP
TFDP1
TFDP2
TINF2
TNIK
TNRC6A
TNRC6B
TNRC6C
TXN
UBA52
UBB
UBC
UBE2C
UBE2D1
UBE2E1
UBE2S
UBN1
VENTX
ALDOC
ENO1
G6PD
GAPDH
HK1
LDHA
PGK1
PKM
PRKAA1
AKT1S1
AMBRA1
ATG10
ATG12
ATG13
ATG14
ATG16L1
ATG3
ATG5
ATG7
BECN1
BMP2
BRAF
CCL3
COL10A1
CXCL1
FKBP8
GABARAP
GABARAPL1
GABARAPL2
GSK3B

IGF1
IL1B
IL24
IL3
IL6R
IL6ST
INHBA
INS
IRF1
KMT2A
LAMP1
LAMP2
MAP1LC3A
MAP1LC3C
MIR29B2
MIR29C
MIR3606
MLST8
MMP14
PCNA
PIK3C3
PLAT
PLAU
RAF1
RB1CC1
RNASEL
SH3GLB1
SLC39A1
SLC39A2
SLC39A3
SLC39A4
SMAD3
SMAD4
SQSTM1
SRC
TGFB1
TNFSF15
ULK1
UVRAG
VTN
FH
GOT1
MDH1
ME1
ME2
PDHA1
PDK1
PDP2
AADAT
ACMSD
AFMID

AHR
EIF2AK1
EIF2AK4
FOXO1
HAAO
IDO1
IDO2
IL1R2
KLF5
KMO
KYNU
NOS1
QPRT
TDO2
TLR4
TNF
CCL27
ELAVL1
GOT2
MDH2
NAMPT
NMNAT2
PARP1
SCO2
SIRT2
SIRT3
SIRT5
SLC2A1
SLC2A4
HTRA1
