# Supplementary material for: An Immunosenescence-Related Gene Signature to Evaluate the Prognosis, Immunotherapeutic Response, and Cisplatin Sensitivity of Bladder Cancer
Source: Dis Markers. 2022 Mar 2;2022:2143892. doi: 10.1155/2022/2143892 (PMC8915927; doi:10.1155/2022/2143892)
Supplement: Supplementary 3 — Supplementary Table 3: the primer sequence utilized in this study. [file 2143892.f3.pdf]

Supplementary Table 3 The primer sequence utilized in this study.

Gene	Sequence (5'-3')
EGFR	Forward:AGGCACGAGTAACAAGCTCAC
	Reverse:ATGAGGACATAACCAGCCACC
TFRC	Forward:ACCATTGTCATATACCCGGTTCA
	Reverse:CAATAGCCCAAGTAGCCAATCAT
ADIPOR2	Forward:CTGGATGGTACACGAAGAGGT
	Reverse:TGGGCTTGTAAGAGAGGGGAC
CTSS	Forward:AAACGGCTGGTTTGTGTGC
	Reverse:CAGTGGTGATCCAGGGTAGG
GBP2	Forward:CTATCTGCAATTACGCAGCCT
	Reverse:TGTTCTGGCTTCTTGGGATGA
PSMD11	Forward:GCCTCCATCGACATCCTCC
	Reverse:GAGCTGCTTTAGCCTTGCTG
KIR2DL4	Forward:GTGGGTTTAACATCTTCACGCT
	Reverse:AGCATCTGTAGGTCTCTCCGT
MAP2K1	Forward:CAATGGCGGTGTGGTGTTC
	Reverse:GATTGCGGGTTTGATCTCCAG
MAPK1	Forward:TACACCAACCTCTCGTACATCG
	Reverse:CATGTCTGAAGCGCAGTAAGATT
IRF1	Forward:ATGCCCATCACTCGGATGC
	Reverse:CCCTGCTTTGTATCGGCCTG
THBS1	Forward:GCCATCCGCACTAACTACATT
	Reverse:TCCGTTGTGATAGCATAGGGG
CCN2	Forward:CAGCATGGACGTTCGTCTG
	Reverse:AACCACGGTTTGGTCCTTGG
SRC	Forward:GAGCGGCTCCAGATTGTCAA
	Reverse:CTGGGGATGTAGCCTGTCTGT
NOX4	Forward:CAGATGTTGGGGCTAGGATTG
	Reverse:GAGTGTTCGGCACATGGGTA
ELAVL1	Forward:GGGTGACATCGGGAGAACG
	Reverse:CTGAACAGGCTTCGTAACTCAT
GAPDH	Forward:GGAGCGAGATCCCTCCAAAAT
	Reverse:GGCTGTTGTCATACTTCTCATGG
