# Supplementary material for: An Immunosenescence-Related Gene Signature to Evaluate the Prognosis, Immunotherapeutic Response, and Cisplatin Sensitivity of Bladder Cancer
Source: Dis Markers. 2022 Mar 2;2022:2143892. doi: 10.1155/2022/2143892 (PMC8915927; doi:10.1155/2022/2143892)
Supplement: Supplementary 4 — Supplementary Figure 1: the expression difference of the 15 genes in the risk signature between adjacent normal and BLCA tissues in the TCGA-BLCA cohort (a), GSE13507 cohort (b), and GSE32894 cohort (c). [file 2143892.f4.pdf]

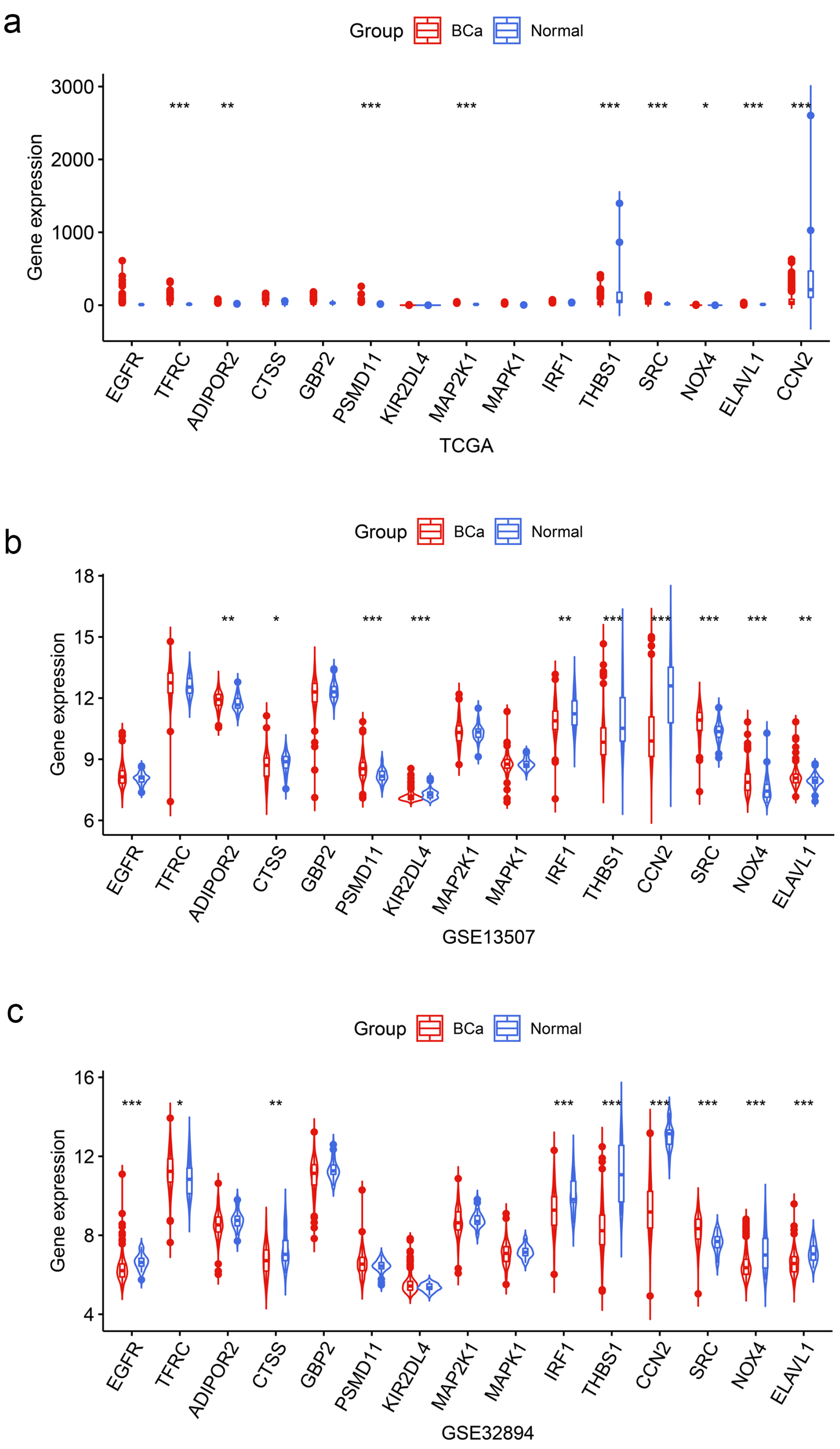

Supplementary Figure 1 The expression difference of the 15 genes in the risk signature between adjacent normal and BCa tissues in the TCGA-BLCA cohort (a), GSE13507 cohort (b), and GSE32894 cohort (c).
