# Supplementary figures and images for: An Immunosenescence-Related Gene Signature to Evaluate the Prognosis, Immunotherapeutic Response, and Cisplatin Sensitivity of Bladder Cancer
Source: Dis Markers. 2022 Mar 2;2022:2143892. doi: 10.1155/2022/2143892 (PMC8915927; doi:10.1155/2022/2143892)

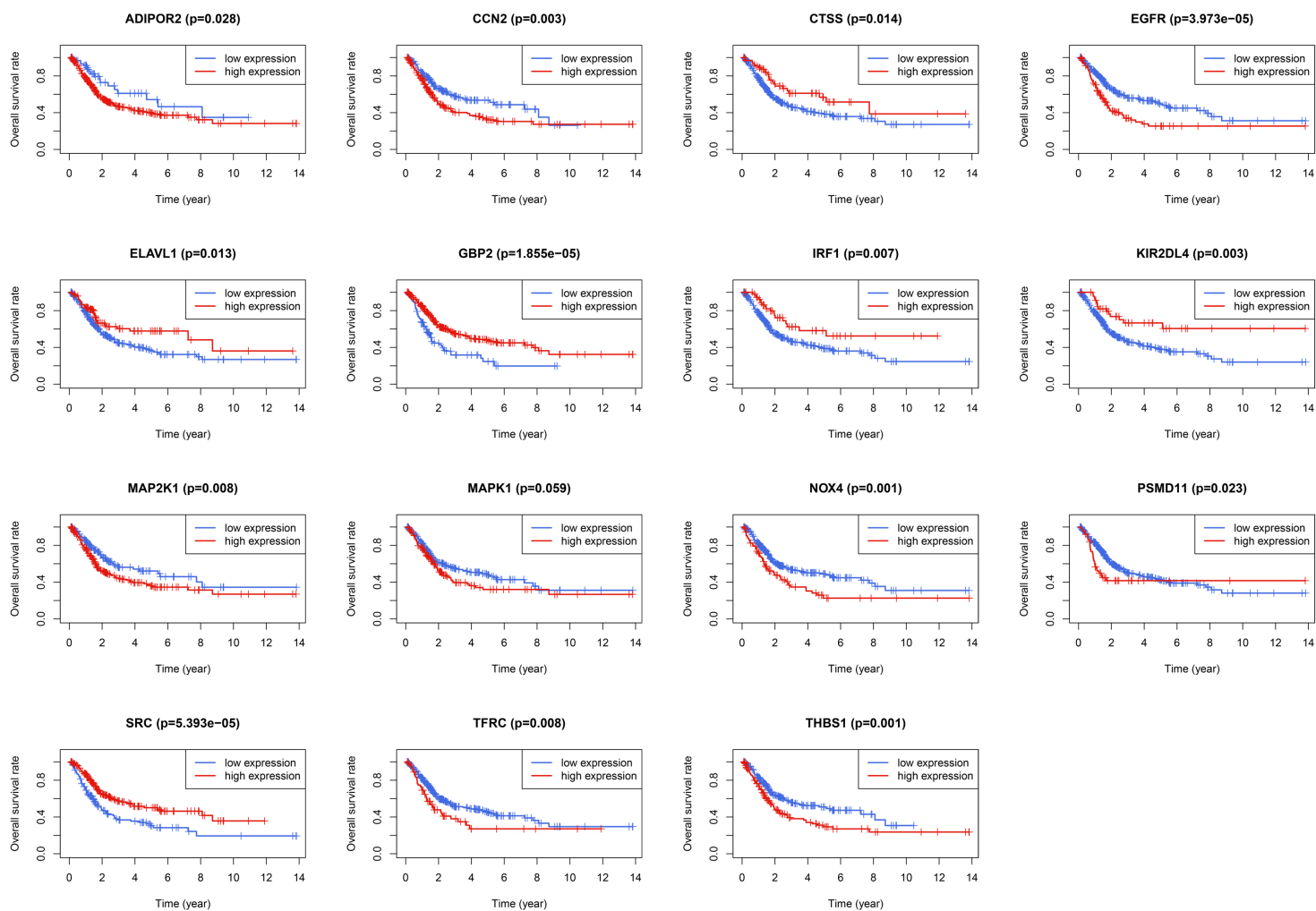

Supplementary Figure 2 The predictive value of the 15 genes to OS in the TCGA-BLCA cohort.

Supplement: Supplementary 5 — Supplementary Figure 2: the predictive value of the 15 genes to OS in the TCGA-BLCA cohort. [file 2143892.f5.pdf]

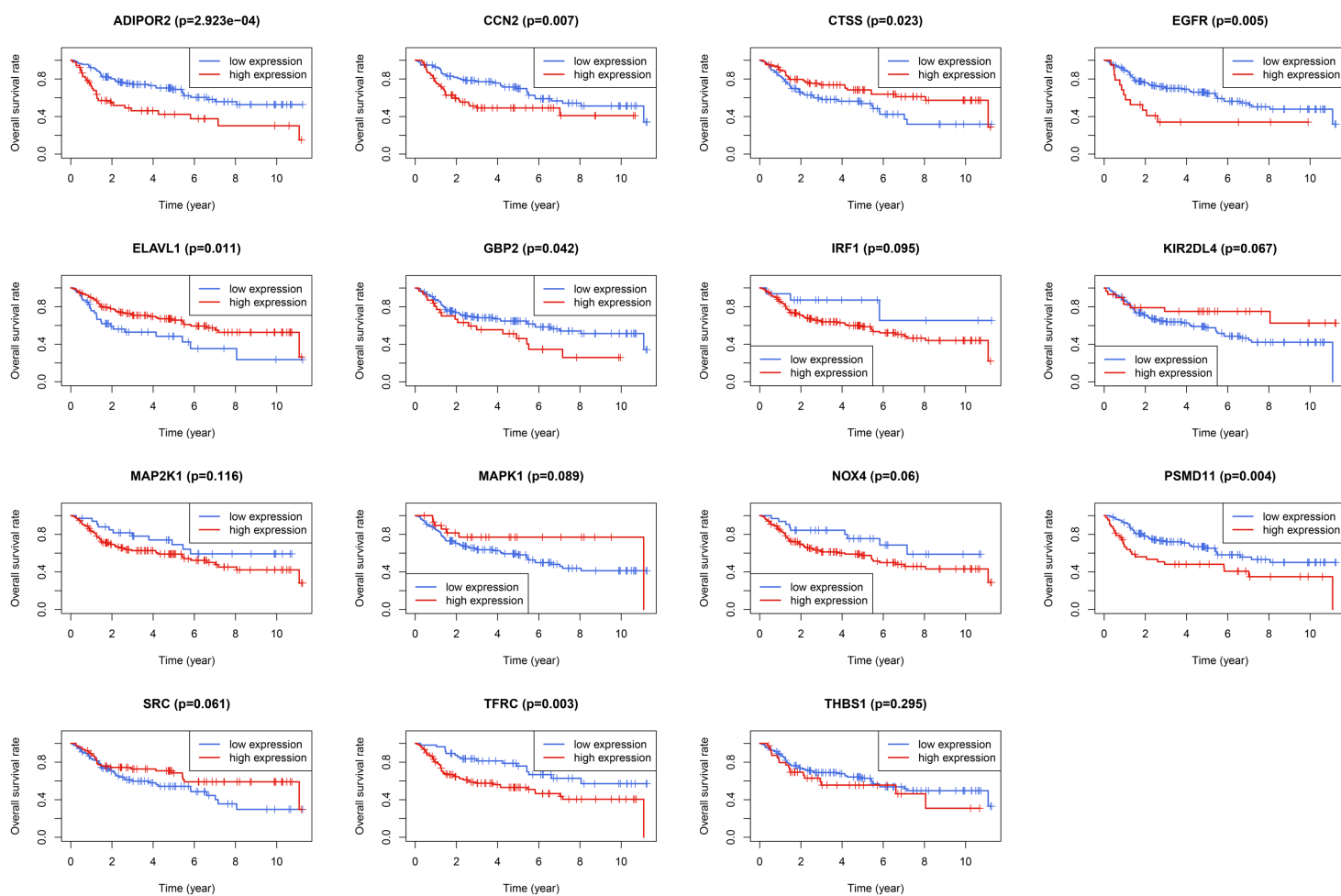

Supplementary Figure 3 The predictive value of the 15 genes to OS in the GSE13507 cohort.

Supplement: Supplementary 6 — Supplementary Figure 3: the predictive value of the 15 genes to OS in the GSE13507 cohort. [file 2143892.f6.pdf]

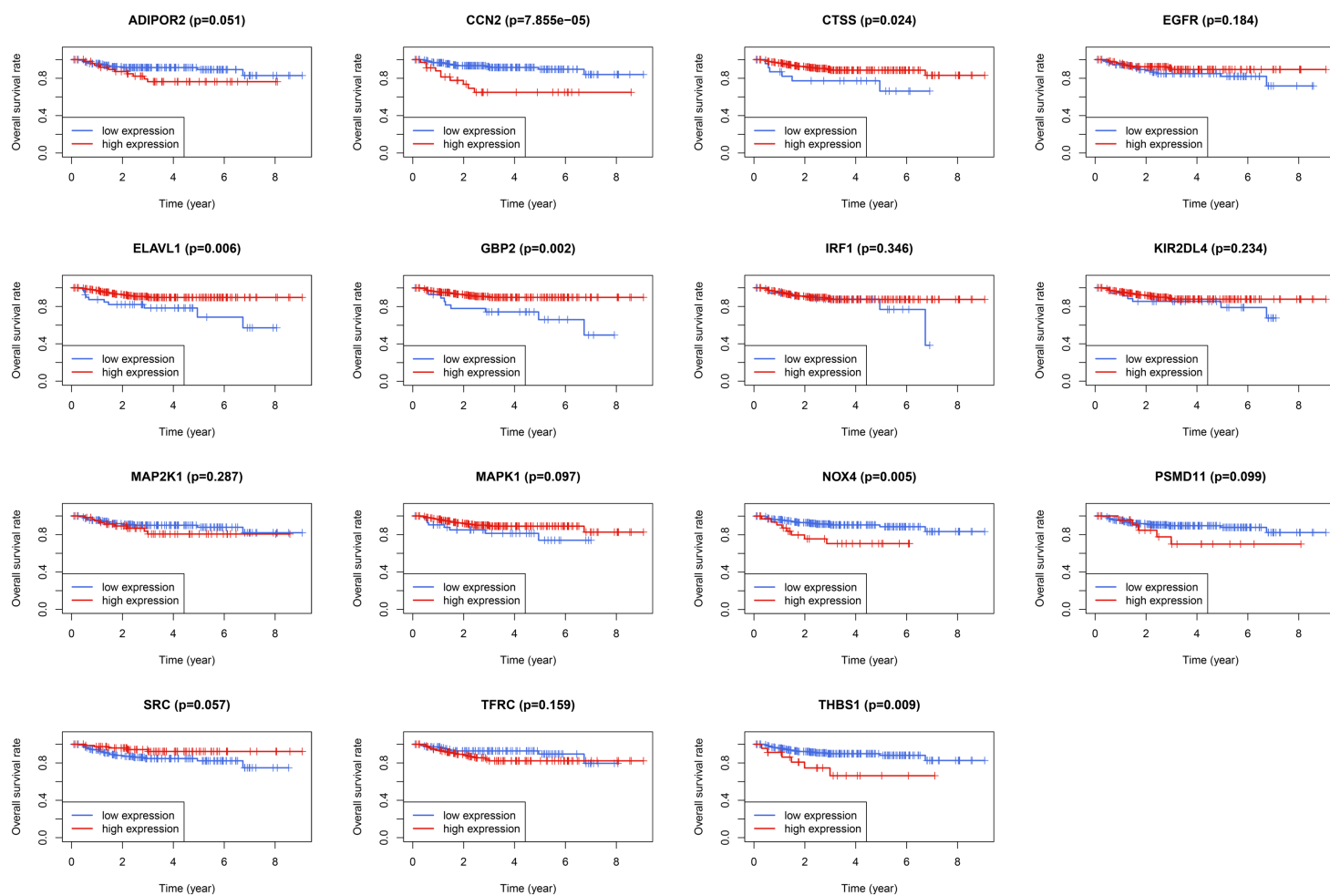

Supplementary Figure 4 The predictive value of the 15 genes to OS in the GSE32894 cohort.

Supplement: Supplementary 7 — Supplementary Figure 4: the predictive value of the 15 genes to OS in the GSE32894 cohort. [file 2143892.f7.pdf]
