# Supplementary material for: An Immunosenescence-Related Gene Signature to Evaluate the Prognosis, Immunotherapeutic Response, and Cisplatin Sensitivity of Bladder Cancer
Source: Dis Markers. 2022 Mar 2;2022:2143892. doi: 10.1155/2022/2143892 (PMC8915927; doi:10.1155/2022/2143892)
Supplement: Supplementary 8 — Supplementary Figure 5: validation of the prognostic value of IRS in the local cohort. (a) The expression level of the 15 genes in the risk signature in the subjects with BLCA in stage I-II and the subjects with BLCA in stage III-IV. (b) The calculated IRS of each patient. (c) The patients in stage III-IV exhibited higher IRS levels compared with those in stage I-II. [file 2143892.f8.pdf]

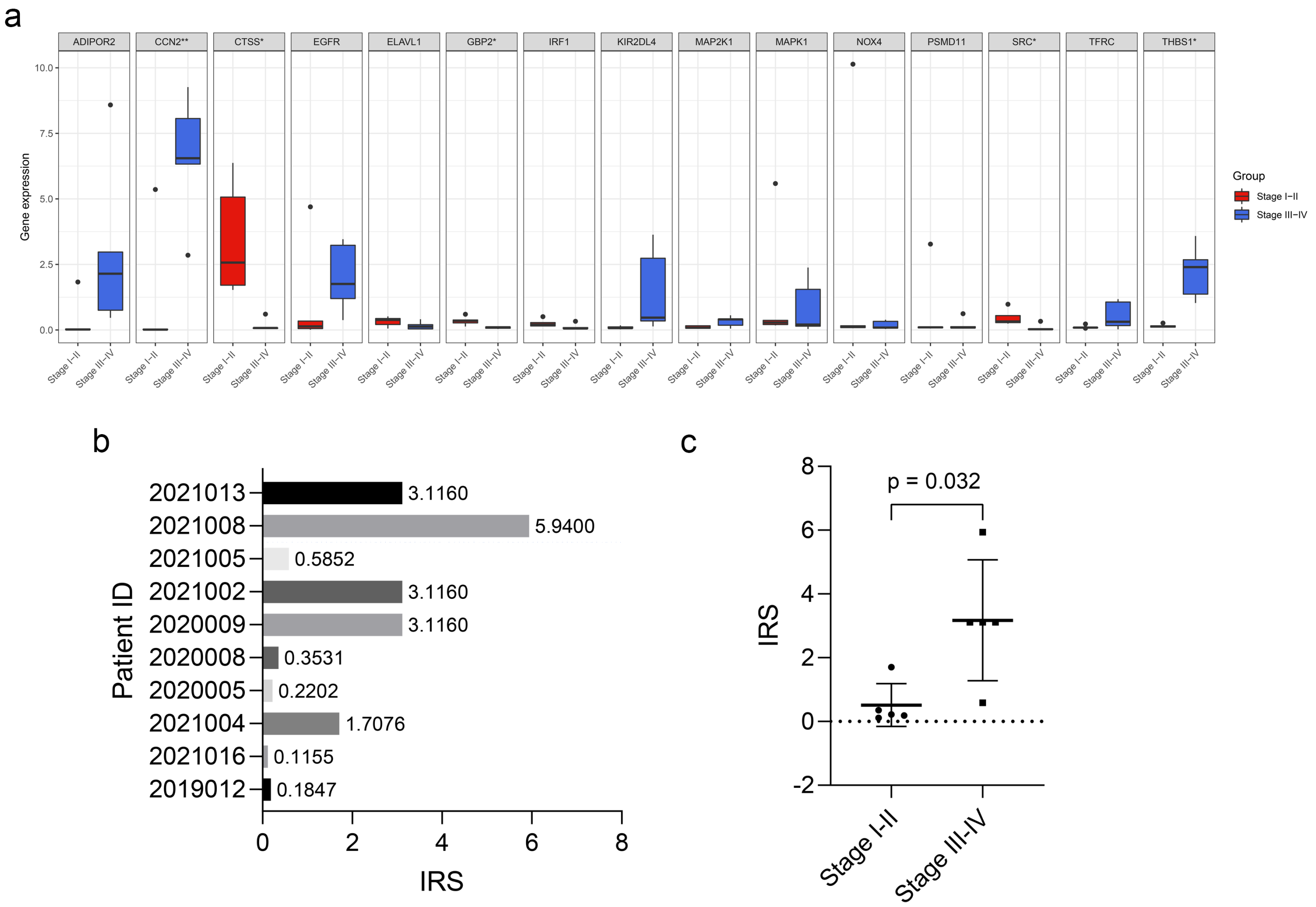


Supplementary Figure 5 Validation of the prognostic value of IRS in the local cohort. (a) The expression level of the 15 genes in the risk signature in the subjects with BCa in stage I-II and the subjects with BCa in stage III-IV. (b) The calculated IRS of each patient. (c) The patients in stage III-IV exhibited higher IRS level compared with those in stage I-II.
