# Supplementary material for: An Immunosenescence-Related Gene Signature to Evaluate the Prognosis, Immunotherapeutic Response, and Cisplatin Sensitivity of Bladder Cancer
Source: Dis Markers. 2022 Mar 2;2022:2143892. doi: 10.1155/2022/2143892 (PMC8915927; doi:10.1155/2022/2143892)
Supplement: Supplementary 9 — Supplementary Figure 6: the expression divergence of the 15 genes in the signature between the patients with high immunotherapeutic sensitivity and the patients with low immunotherapeutic sensitivity (a) and the prognostic value of each gene in the IMvigor210 cohort (b). [file 2143892.f9.pdf]

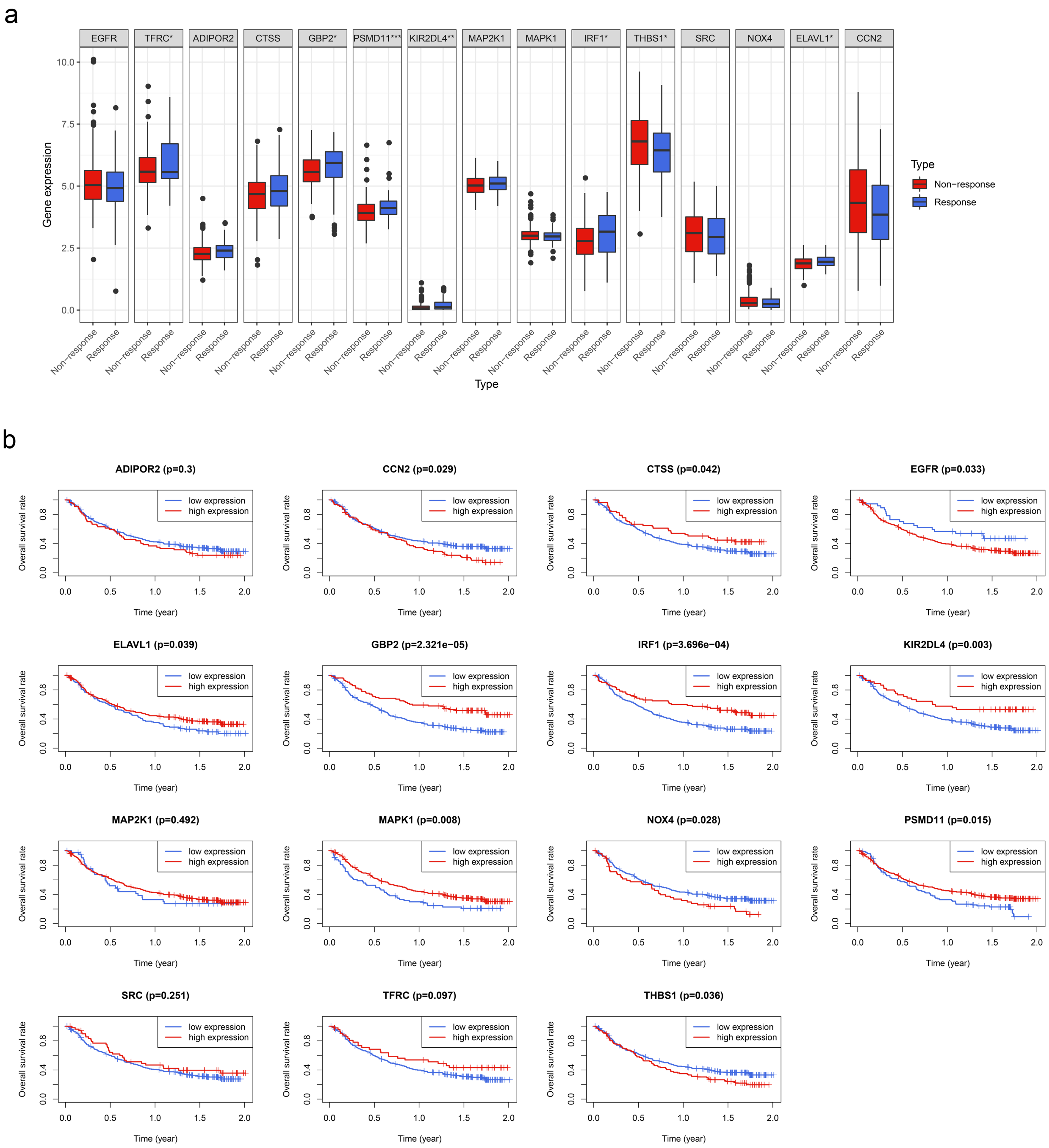

Supplementary Figure 6 The expression divergence of the 15 genes in the signature between the patients with high immunotherapeutic sensitivity and the patients with low immunotherapeutic sensitivity (a) and the prognostic value of each gene in the IMvigor210 cohort (b).
